# Analysis of m6A-Related Signatures in the Tumor Immune Microenvironment and Identification of Clinical Prognostic Regulators in Adrenocortical Carcinoma

**DOI:** 10.3389/fimmu.2021.637933

**Published:** 2021-03-03

**Authors:** Yi Jin, Zhanwang Wang, Dong He, Yuxing Zhu, Xueying Hu, Lian Gong, Mengqing Xiao, Xingyu Chen, Yaxin Cheng, Ke Cao

**Affiliations:** ^1^Department of Oncology, Third Xiangya Hospital, Central South University, Changsha, China; ^2^Department of Radiation Oncology, Hunan Cancer Hospital, The Affiliated Cancer Hospital of Xiangya School of Medicine, Central South University, Changsha, China; ^3^Key Laboratory of Translational Radiation Oncology, Department of Radiation Oncology, Hunan Cancer Hospital and The Affiliated Cancer Hospital of Xiangya School of Medicine, Central South University, Changsha, China; ^4^Department of Respiratory, The Second People's Hospital of Hunan Province, Changsha, China

**Keywords:** adrenocortical carcinoma, M6A, tumor immune microenvironment, prognostic signatures, HNRNPA2B1

## Abstract

Adrenocortical carcinoma (ACC) is a rare endocrine malignancy with a high rate of mortality and recurrence. N6-methyladenosine methylation (m6A) is the most common modification to affect cancer development, but to date, the potential role of m6A regulators in ACC prognosis is not well understood. In this study, we systematically analyzed 21 m6A regulators in ACC samples from The Cancer Genome Atlas (TCGA) and the Gene Expression Omnibus (GEO) database. We identified three m6A modification patterns with different clinical outcomes and discovered a significant relationship between diverse m6A clusters and the tumor immune microenvironment (immune cell types and ESTIMATE algorithm). Additionally, Gene Ontology (GO), Kyoto Encyclopedia of Genes and Genomes (KEGG), and Gene Set Enrichment Analysis (GSEA) revealed that the m6A clusters were strongly associated with immune infiltration in the ACC. Next, to further explore the m6A prognostic signatures in ACC, we implemented Lasso (Least Absolute Shrinkage and Selection Operator) Cox regression to establish an eight-m6A-regulator prognostic model in the TCGA dataset, and the results showed that the model-based high-risk group was closely correlated with poor overall survival (OS) compared with the low-risk group. Subsequently, we validated the key modifications in the GEO datasets and found that high HNRNPA2B1 expression resulted in poor OS and event-free survival (EFS) in ACC. Moreover, to further decipher the molecular mechanisms, we constructed a competing endogenous RNA (ceRNA) network based on HNRNPA2B1, which consists of 12 long noncoding RNAs (lncRNAs) and 1 microRNA (miRNA). In conclusion, our findings indicate the potential role of m6A modification in ACC, providing novel insights into ACC prognosis and guiding effective immunotherapy.

## Introduction

Adrenocortical carcinoma (ACC) is an uncommon endocrine malignancy with an annual incidence of 0.7–2.0 cases per million (1). Despite its rarity, the 5-year survival rate in most series is <35% (2). Currently, the only curative therapy for localized ACC is surgery. Even with complete excision, rates of local recurrence typically range from 19–34% (3, 4). Adjuvant treatments which aim to decrease recurrence, including chemotherapy and radiotherapy, show limited therapeutic effectiveness (5). Nevertheless, the most widely used tumor, lymph node, and metastasis (TNM) classification system remains unacceptable for heterogeneous outcomes and poor survival (6, 7). Therefore, unraveling the genomic properties underlying ACC is crucial for developing effective treatments and predicting individual survival and recurrence risk.

N6-methyladenosine (m6A), which was first discovered in the 1970s, is recognized as the most prominent and abundant form of internal modification that occurs in messenger RNAs (mRNAs) and long non-coding RNAs (lncRNAs) in many eukaryotic species (8, 9). m6A methylation is thought to affect every aspect of RNA metabolism, including RNA splicing, translocation, stability, and translation into protein (10). The m6A modification is dynamically deposited by three types of homologous factors: methyltransferases (“writers”), demethylases (“erasers”), and m6A binding proteins (“readers”) (11). Methyltransferases, with core members, METTL3, METTL14, WTAP, ZC3H13, RBM15, and RBM15B, catalyze the methyl group directly attached to the nitrogen on the sixth carbon of the aromatic ring of an adenosine residue (12). Demethylases, which mediate the m6A removal process, with core members FTO and ALKBH5, selectively remove the methyl code from specific mRNAs (11). m6A binding proteins, including the YTHDF family (YTHDF1/2/3), nuclear heterogeneous riboprotein family (HNRNPA2B1 and HNRNPC), and eukaryotic initiation factor (eIF or EIF1A), aim to decode RNA methylation and recognize the m6A motif (11).

To date, N6-methyladenosine, a potential biomarker, has been reported to actively participate in various important physiological processes such as stem cell differentiation, circadian periods, and DNA damage response *in vivo* (13–15). Aberrant expression and mutation of m6A were confirmed to result in the abnormal processes, including dysregulation of cell death and proliferation, developmental defects, and impaired self-renewal capacity (16, 17). Recent studies have demonstrated that abnormal m6A methylation modification is closely associated with a variety of human diseases, especially cancer, including bladder cancer, head and neck squamous cell carcinoma, gastric cancer, breast cancer, hepatocellular carcinoma, and colorectal cancer (18). For instance, in breast cancer, high FTO levels are significantly associated with poor survival rates. Furthermore, in a series of *in vitro* and *in vivo* assays, FTO dramatically alleviated and degraded BNIP3 (a tumor suppressor) *via* a YTHDF2-independent mechanism to induce cancer cell proliferation, colony formation, and metastasis (19). Additionally, Zewei Tu reportedly built a ceRNA network and established a 9 m6A-related lncRNA prognostic model in lower-grade glioma patients (20). Accumulating evidence has shown that m6A-related mRNAs and lncRNAs can serve as novel potential targets for predicting prognosis and developing personalized treatments for many types of cancer. However, little is known about the relationship between the effect of m6A methylation modification and ACC.

The tumor microenvironment (TME), which includes cancer cells, stromal cells, and distant recruited cells, such as infiltrating immune cells (myeloid cells and lymphocytes), bone marrow-derived cells (BMDCs), and secreted factors such as cytokines and chemokines, play a crucial role in tumor progression and affect the clinical benefit from novel strategies of immunological checkpoint blockade (ICB, PD-1/L1, and CTLA-4) (21, 22). In advanced ACC, no investigated therapy has offered long-term disease control, except for immune checkpoint blockade. A phase II study indicated that pembrolizumab (an anti-PD-1 monoclonal antibody) can provide meaningful and durable antitumor activity (23). Emerging studies have focused on research interests that enhance the in-depth understanding of the heterogeneity and complexity of the TME to improve immunotherapy strategies by comprehensive analysis of particular m6A regulators (24, 25). For instance, inhibiting ALKBH5 may enhance the efficacy of anti-PD-1 therapy in melanoma patients by mediating the level of Mct4/Slc16a3, which is involved in regulating suppressive lymphocyte Treg and myeloid-derived suppressor cell accumulations in the TME (24). Currently, none of these studies have extended their research into the frontiers of knowledge in ACC.

In this study, we performed a retrospective analysis based on The Cancer Genome Atlas (TCGA) and the Gene Expression Omnibus (GEO) databases to estimate the effect of m6A-related genes on the prognostic value. We identified multiple m6A regulators and related lncRNAs or microRNAs (miRNAs) as potential biomarkers by shaping individual TME characterizations.

## Materials and Methods

### Data Processing of the ACC Dataset

The public RNA sequencing, mutation expression, and full clinical information of ACC were downloaded from TCGA and GEO. Patients without survival information were excluded from further evaluation. The RNA sequencing data (FPKM value) and somatic mutation data from TCGA-ACC (The Cancer Genome Atlas - Adrenocortical carcinoma) were downloaded from the Genomic Data Commons (GDC; https://portal.gdc.cancer.gov/) and gathered as a training set for further analysis. In total, six eligible data from GEO (GSE10927, GSE19750, GSE33371, GSE76019, GSE76021, and GSE49280) were downloaded and an averaging method with the affy and simpleaffy packages was used to perform background adjustment and quantile normalization.

### Consensus Clustering of m6A Regulators

We first selected 21 m6A RNA methylation regulators from previously published articles (26, 27). These 21 m6A regulators included 8 writers (METTL3, METTL14, RBM15, RBM15B, WTAP, KIAA1429, CBLL1, and ZC3H13), 2 erasers (ALKBH5, FTO), and 11 readers (YTHDC1, YTHDC2, YTHDF1, YTHDF2, YTHDF3, IGF2BP1, HNRNPA2B1, HNRNPC, FMR1, LRPPRC, and ELAVL1). Based on the expression of the 21 m6A modulators, the patients were classified into three groups using the optimal k-means clustering (“kmeans” function in R). Cluster analysis was performed using the ConsensusClusterPlus R package with cycle computation 1,000 times to ensure stability and reliability (28). The overall survival (OS) between different clusters was calculated using the Kaplan-Meier method.

### Identification of Differentially Expressed Genes (DEGs) Between m6A Patterns

To identify the DEGs between three clusters in the TCGA-ACC cohort, the empirical Bayesian approach of the limma R package was applied in the standard comparison mode. The significance criteria for determining DEGs was set to | logFC | > 1 and *P*-value < 0.05. To investigate the pathways enriched in the different subgroups, we performed Kyoto Encyclopedia of Genes and Genomes (KEGG) pathway analysis and Gene Ontology (GO) biological processes by applying a threshold *P*-value < 0.05, minimum count of 5, and enrichment factor > 0.15. Gene set enrichment analysis (GSEA) was used to evaluate all genes based on their log2 fold change and assess the functions associated with subtypes by implementing the clusterProfiler R package.

### Comparison of Immune Cell Infiltration Among m6A Patterns

To explore the degree of immune cell infiltration among the three subgroups, we applied the ESTIMATE algorithm, in which R script was downloaded from the website (https://sourceforge.net/projects/estimateproject/) to calculate the estimate scores, immune scores, and stromal scores for further predicting tumor purity and analyzing the TME (29). To explore the differences in immune cell subtypes among multiple clusters, we utilized the CIBERSORT package to assess the proportions of 22 immune cell subtypes based on TCGA-ACC samples. The results with *P* < 0.05 in CIBERSORT analysis were used for further analysis. The Mann-Whitney U test was used to compare the differences among the three subgroups.

### Least Absolute Shrinkage and Selection Operator Cox Regression and Validation of the Prognostic m6A Signatures

To enhance the prediction accuracy and interpretability of the statistical model, Lasso Cox regression analysis was carried out to examine the relationship between m6A prognosis signatures and ACC risk. Using the “glmnet” software package of R, 8 m6A-related genes were screened to construct the best prognostic model. The risk score was generated using the following formula: risk score = Expression_mRNA1_ × Coefficient_mRNA1_ + Expression_mRNA2_ × Coefficient_mRNA2_ +…Expression_mRNAn_ × Coefficient_mRNAn_. According to the predictive model, the patients were divided into high-risk and low-risk groups using the median cutoff of risk score. The Cox proportional hazard regression model includes age and TNM stage. The hazard ratio (HR) from Cox regression analysis was used to distinguish the prognostic factors positively or negatively. A gene with HR > 1 was considered a risk gene, and a gene with HR < 1 was considered a protective gene. Subsequently, the Kaplan-Meier survival method was used to evaluate the availability of the prognostic model, and the sensitivity and specificity of the receiver operating characteristic (ROC) curve were used to evaluate the prognostic accuracy of the signature building. Similarly, the five validated GEO cohorts were further calculated to validate the prognostic value of selected m6A-related genes by OS and PFS (progression-free survival) analyses.

### Gene Mutation Screening and Analysis

The SNP dataset was based on VarScan2 variant aggregation and masking data in TCGA. Here, we analyzed the SNP mutation and carried out visualization using the R maftools package (30). The SNP2APA database was designed to explore the effects of single nucleotide polymorphisms (SNPs) and provided OS across different cancer types (31). Based on this database, we searched for specific SNPs that have prognostic value in ACC, and assessed the relationship between these SNPs and key m6A-related genes.

### Construction of the ceRNA Network

Using the TCGA cohort, differentially expressed genes between m6A patterns were identified with the standards of | log2(Fold change) | > 1 and *P* < 0.05 using the R package “limma.” Perl programming language was applied to target miRNAs-lncRNAs and miRNAs-mRNAs in the prediction analysis. Furthermore, miRcode was used to collect and target experimentally validated lncRNAs (32). StarBase v3.0 was used to predict miRNA-mRNA interactions (http://starbase.sysu.edu.cn/) (33). The ceRNA network was visualized using the “Cytoscape” software (34).

### Statistical Analysis

Most analyses were performed using R software (version 3.6.1, http://www.R-project.org). Kaplan-Meier curves and the log-rank test were used to compare the OS between various subgroups based on the expression of m6A-related genes. Univariate and multivariate Cox proportional hazard regression analyses were used to evaluate the independent prognostic value of the clinical characteristics of OS. The prognostic ability of the predictive models for 1/3/5-year OS was evaluated by ROC curves (R package “timeROC”) and the area under the curve (AUC) values. In all analyses, all statistical *P*-values were bilateral, and *P* < 0.05 was considered statistically significant.

### Clinical Specimens

Adrenocortical carcinoma tissues and normal adrenocortical tissues were obtained from five patients who received operation at the Third Xiangya Hospital (Changsha, Hunan, China) from January 2016 to December 2020. The patients were diagnosed by pathological analysis and were not subjected to chemotherapy or radiotherapy. The Institutional Review Board of the Ethics Committee of Third Xiangya Hospital approved the consent procedure, and written informed consent was provided by all patients in this research.

### Immunohistochemistry

Paraffin-embedded ACC tissues and normal tissues were sliced, dewaxed, hydrated, and antigen-repaired, then endogenous peroxidase was blocked; anti-HNRNPA2B1 (1:100, 14813-1-AP, Proteintech Group, Wuhan, China), anti-LRPPRC (1:100, 21175-1-AP, Proteintech Group), and anti-ELAVL1 (1:100, 11910-1-AP, Proteintech Group) were added and incubated them together at 4°C overnight, respectively. Polymer enhancers were incubated for 30 min at room temperature, then biotinlabeled secondary antibodies were added and incubated for 30 min at room temperature. Next, the sections were stained by using diaminobenzidine staining solution, followed by counterstaining with hematoxylin, and then the sections were mounted in glycerol-vinyl-alcohol. Two independent professional pathologists were blinded to analysis the data and histopathological features of the patients, and also evaluated the IHC scores according to the scoring standards.

## Results

### Consensus Clustering of m6A Genes in Three Clusters With Different Clinical Outcomes of ACC

**Figure 1:**
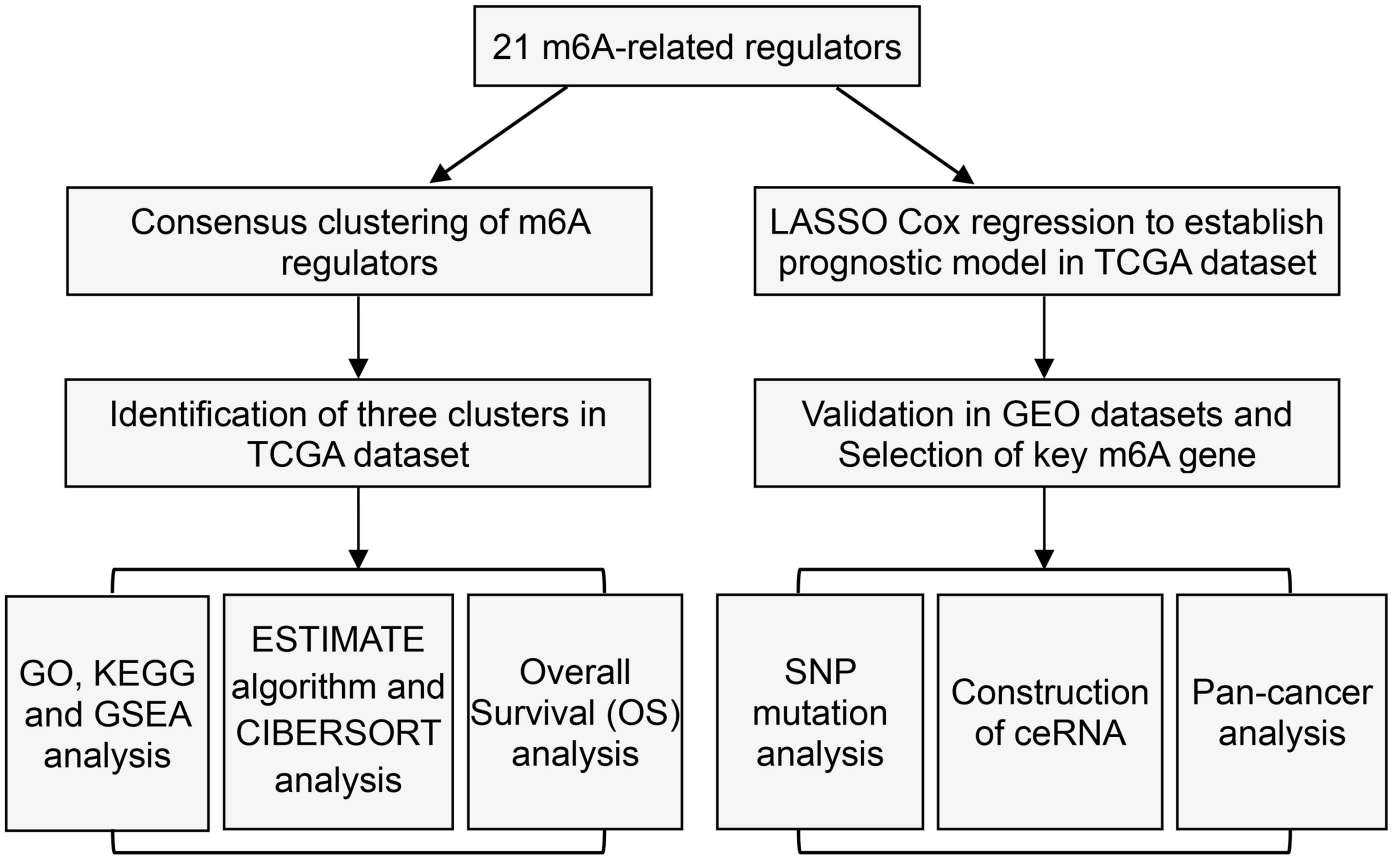
Study flow chart.

**Figure 2:**
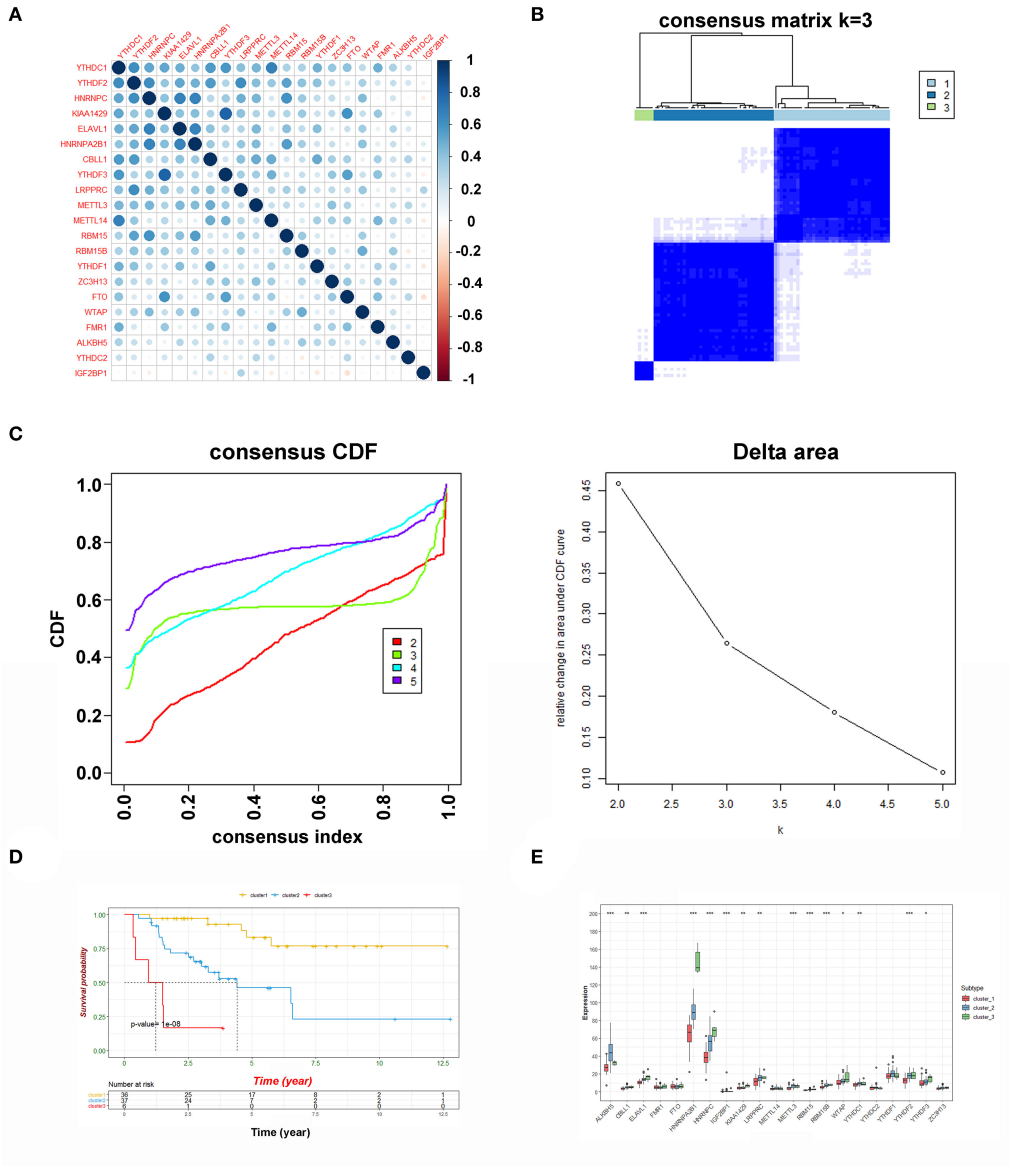
Consensus clustering of m6A genes. **(A)** The Pearson correlation analysis was used to search the relationship among m6A regulators. **(B)** Consensus clustering matrix for k = 3. **(C)** Consensus clustering cumulative distribution function (CDF) and relative change in area under CDF curve for k = 2 to 5. **(D)** Kaplan-Meier curves of OS for three clusters in ACC. **(E)** The expression of the 21 m6A regulatory factors in clusters (***P < 0.001; **P < 0.01; *P < 0.05).

Here, the clinical data and corresponding gene expression profiles of ACC patients were downloaded from the TCGA and GEO databases. The workflow is shown in Figure 1. We first analyzed 21 m6A regulators and mapped the correlation between m6A patterns and ACC survival. As a result, the expression levels of m6A regulators and clinical characteristics were obtained from TCGA and GEO. Pearson correlation analysis was performed to determine the relationship between m6A regulators (Figure 2A). Some highly correlated (|correlation coefficient| ≥ 0.5, *P* < 0.05) m6A regulators were identified, such as YTHDF1, YTHDF2, HNRNPC, KIAA1429, ELAVL1, HNRNPA2B1, CBLL1, and YTHDF3. Based on the ConsensusClusterPlus R package, the TCGA-ACC cohort was clustered into different groups by consensus expression of m6A regulators. When the consensus matrix k value was equal to 3, there was the least crossover between the ACC samples (Figures 2B,C and Supplementary Figure 1). The OS difference between different clusters was calculated using the Kaplan-Meier method. Significantly better OS was found in patients specifically in cluster1 compared with other clusters (Figure 2D). Further, we plotted a boxplot (Figure 2E) and heatmap (Figure 4A) for visualizing the expression of the 21 m6A regulatory factors in clusters and found that the expression of CBLL1, ELAVL1, HNRNPA2B1, HNRNPC, KIAA1429, LRPPRC, RBM15, RBM15B, WTAP, YTHDF2, and YTHDF3 in cluster3 was higher than that in other clusters (*P* < 0.01), while the expression of ALKBH5, IGF2BP1, METTL3, and YTHDF1, was higher in cluster2 than in other clusters.

### The Interaction and Correlation Among the m6A Regulators in Three Patterns

**Figure 3:**
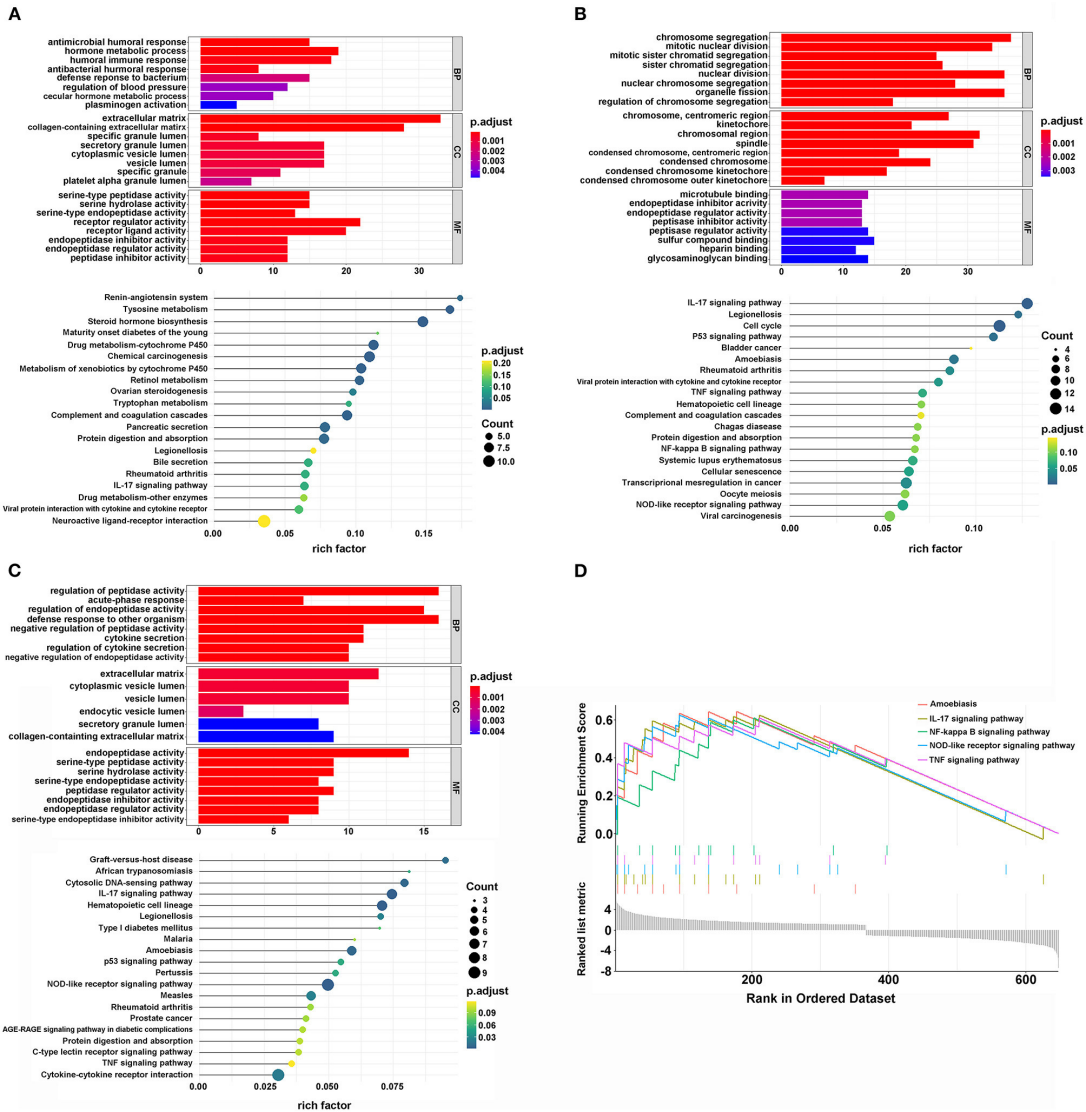
The interaction and correlation among clusters. **(A)** The GO and KEGG analysis of DEGs between cluster1 and cluster2. **(B)** The GO and KEGG analysis of DEGs between cluster1 and cluster3. **(C)** The GO and KEGG analysis of DEGs between cluster2 and cluster3. **(D)** The GSEA analysis of DEGs between cluster1 and cluster2.

To explore the potential biological differences among the three different m6A modification patterns, we identified DEGs by comparing clusters with the threshold of |logFC (fold change)| ≥ 1 and adj. *P* < 0.05. When comparing cluster1 and cluster2, there were 371 up-regulated and 292 down-regulated genes. GO analysis of biological processes showed that the DEGs were enriched in extracellular structure organization, matrix organization, and humoral immune response. Cellular component analysis indicated that DEGs were abundant in the extracellular matrix and collagen-containing extracellular matrix. Molecular function analysis indicated that DEGs were mainly located in receptor regulator and ligand activities. Furthermore, KEGG analysis showed that the DEGs were enriched in tyrosine metabolism, viral protein interaction with cytokine and cytokine receptor, and IL-17 signaling pathways (Figures 3A–C). Additionally, GSEA was performed for further signaling pathway enrichment analysis, and in a comparison between clusters1 and cluster2, the IL-17 signaling pathway, TNF, and NF-kappa B signaling pathway were enriched relative to cluster2 (Figure 3D). These signaling pathways are related to core biological carcinogenic processes, most of which are involved in the regulation of immune checkpoint expression, and have potential for further exploration of the effect of m6A modifications on immunotherapy (25, 35, 36).

### Immune Landscape in ACC Patients

**Figure 4:**
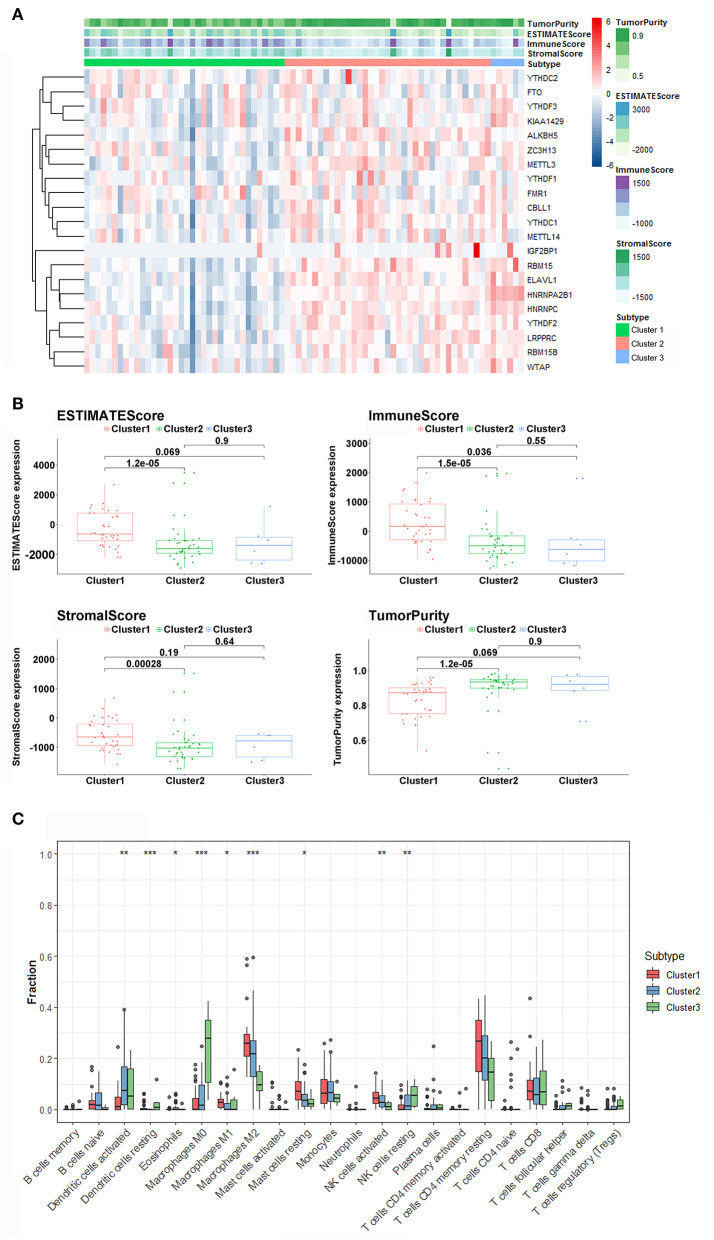
Immune characteristics among three m6A patterns. **(A)** The heatmap of m6A regulators from 3 clusters and ESTIMATE algorithm. **(B)** Different expression of ESTIMATE score, immune score, stromal score, and tumor purity in three m6A Patterns. **(C)** Differences in the levels of infiltration of the 22 immune cells in three m6A patterns (***P < 0.001; **P < 0.01; *P < 0.05).

The ESTIMATE algorithm provided stromal and immune scores, and tumor purity for all ACC samples. The heatmap of m6A-related gene expression and the stromal, immune, and ESTIMATE scores, and tumor purity are shown and clustered in Figure 4A. From the clustering, we found that such m6A genes showed a similar expression trend with tumor purity, and an opposite trend with stromal, immune, and ESTIMATE scores, indicating that the m6A pathway plays an important role in the tumor immune microenvironment and determines tumor progression and metastatic dissemination. Moreover, the density and location of immune cells can be quantified as a tangible indicator by an immune score. Here, there was a significant difference in the immune scores between the m6A clusters, and cluster1 showed the highest immune score. Furthermore, the ESTIMATE and stromal scores were also calculated, and the expression of cluster1 was higher than that of cluster2. Conversely, the distribution of tumor purity was different from the stromal, immune, and ESTIMATE scores, and cluster1 showed a lower tumor purity score than the others (Figure 4B).

The 22 different immune cell types among different clusters were analyzed using the CIBERSORT algorithm. The results revealed that the macrophages M0, MI, M2 macrophages, dendritic cells activated, dendritic, cells resting, eosinophils mast cells resting, natural killer (NK) cells activated, and NK cells resting accounted for a large proportion of immune cell infiltration. Furthermore, cluster1, which had better survival, displayed a greater number of M1, M2 macrophages, and NK cells activated compared with other clusters with worse prognosis (Figure 4C). In addition, we found that the levels of dendritic cells, macrophages M0, and NK cells activated in cluster1 were significantly lower than those in cluster2 and cluster3. The outcome revealed that m6A-related patterns may remarkably suppress or strengthen the expression of specific immune cell types, thus potentially influencing the response to immunotherapy.

### Prognostic Analysis of Risk Model and m6A Genes

**Figure 5:**
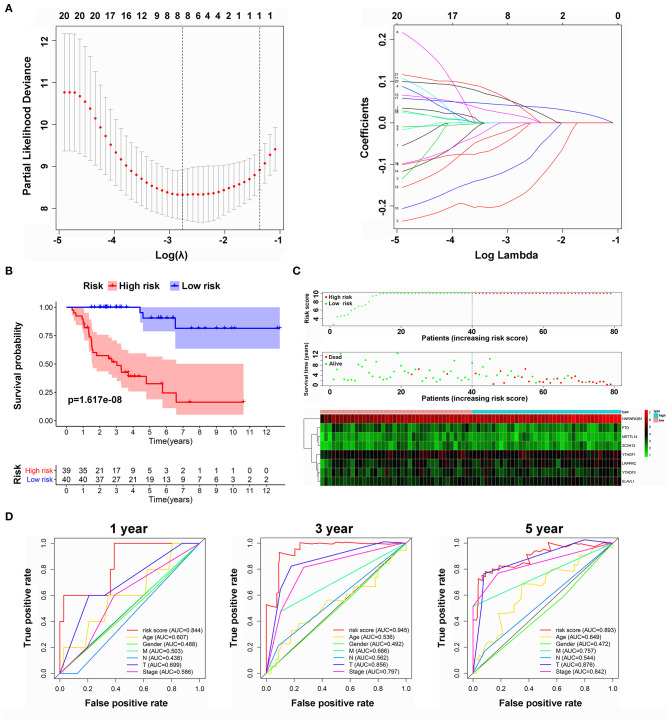
Risk model from m6A-related genes. **(A)** Lasso Cox regression analysis of 21 m6A-related genes. **(B)** Overall survival analysis for patients in high/low risk. **(C)** The distributions of risk scores, alive/dead status, and expression of three m6A-related genes. **(D)** The ROC curve of risk score and clinical characteristics.

To develop a signature for prognosis prediction of ACC, we performed Lasso Cox regression analysis on 21 m6A-related genes based on the TCGA database. Next, we obtained eight genes (METTL14, ZC3H13, FTO, YTHDF1, YTHDF3, HNRNPA2B1, LRPPRC, and ELAVL1) to build the risk model, and the coefficients of these genes were used to calculate the risk score (Figure 5A). The risk score = METTL14 × (−0.1750) + ZC3H13 × (−0.0212) + FTO × (−0.0984) + YTHDF1 × 0.0159 + YTHDF3 × (−0.0073) + HNRNPA2B1 × 0.0405 + LRPPRC × 0.0437 + ELAVL1 × 0.0376. Patients with ACC were separated into low-risk or high-risk groups with the median cutoff of risk score. As shown in Figures 5B,C, we found that as the risk score increased, high-risk patients had significantly worse OS than low-risk patients (*P* = 1.617e^−08^). Univariate and multivariate analyses were used to evaluate the prognostic value of age, sex, M, N, T, and clinical stage (Supplementary Figure 2); however, only T stage was significantly correlated with OS. The ROC curve showed that the risk score had strong predictive ability, with an AUC of 0.844, 0.945, and 0.893 in 1, 3, and 5 years compared with other factors (Figure 5D). These results indicate that the risk model may serve as an important indicator for evaluating the prognosis of ACC.

### Validation of the m6A-Genes in the GEO Dataset

**Figure 6:**
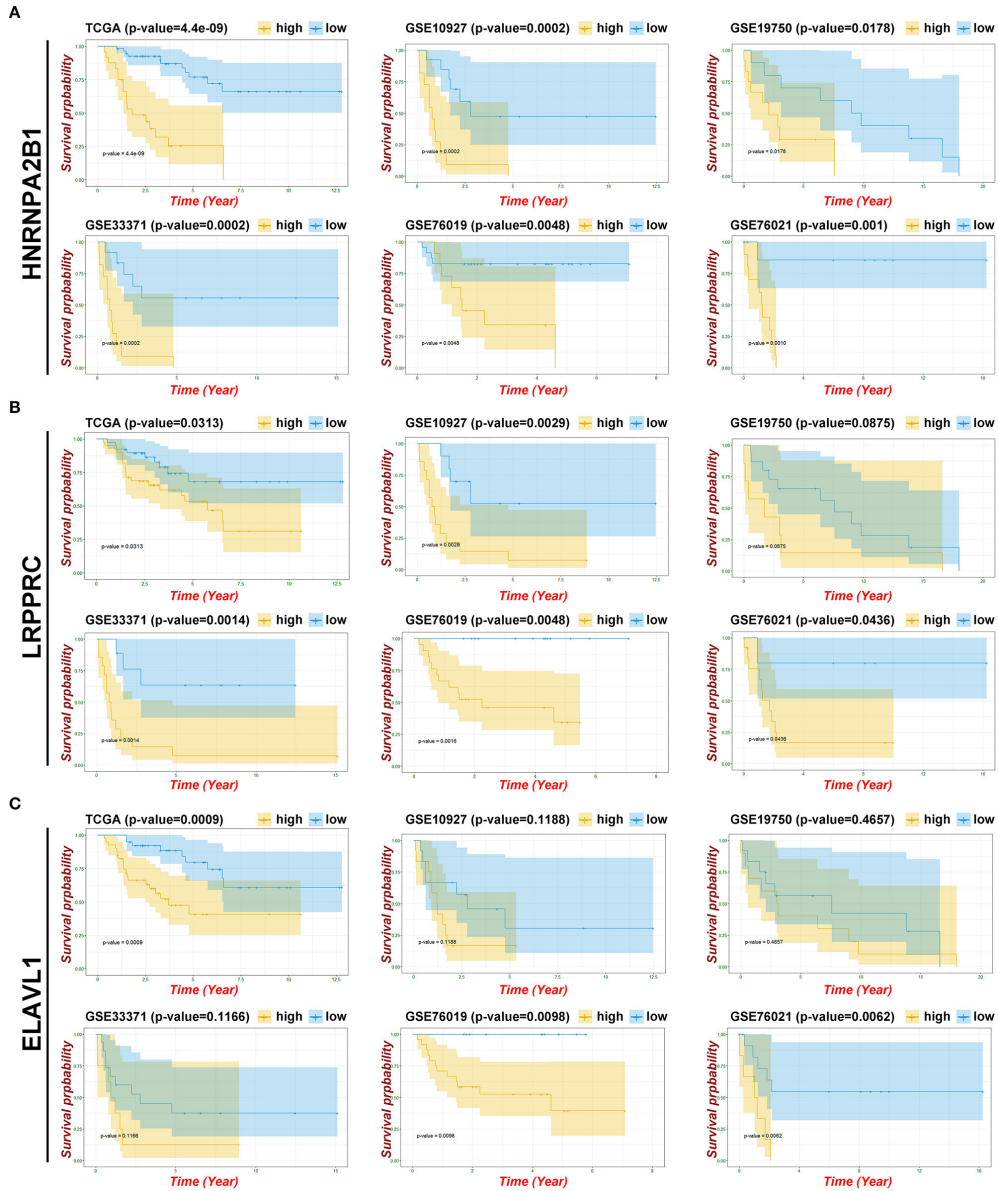
Identifying the key gene of m6A patterns. **(A)** Kaplan-Meier OS curves and EFS curves for patients in HNRNPA2B1. **(B)** Kaplan-Meier OS curves and EFS curves for patients in LRPPRC. **(C)** Kaplan-Meier OS curves and EFS curves for patients in ELAVL1.

To verify and identify the key genes of m6A patterns, we evaluated the prognostic values of the eight genes and the risk model in the GEO datasets. Three datasets (GSE10927, GSE19750, and GSE33371) containing OS statistics and two datasets (GSE76019 and GSE76021) containing event-free survival (EFS) statistics were selected for further validation. Fortunately, the results indicated that the risk model have potential value of prediction in all GEO datasets (Supplementary Figure 3). Considering the similarities of identified genes in the TCGA and GEO data, it is believed that the overlapping m6A regulators might be significant, including HNRNPA2B1, LRPPRC, FTO, YTHDC1, and ELAVL1 (Supplementary Table 1). HNRNPA2B1 was validated as a significant indicator of poor OS and EFS based on all GEO datasets (Figure 6A). LRPPRC might be regarded as a crucial biomarker of poor survival, which was successfully validated on four GEO datasets (GSE10927, GSE33371, GSE76019, and GSE76021) (Figure 6B). Furthermore, ELAVL1 was re-verified as a potential biomarker based on two GEO containing EFS data (Figure 6C). However, other m6A-related genes received a failed verification on most GEO datasets (Supplementary Figures 3, 4). Subsequently, compared with the normal tissue, the expression of these three m6A regulators was upregulated in ACC by performing IHC (Supplementary Figure 5).

### SNP Analysis of m6A Genes Among Three Patterns

**Figure 7:**
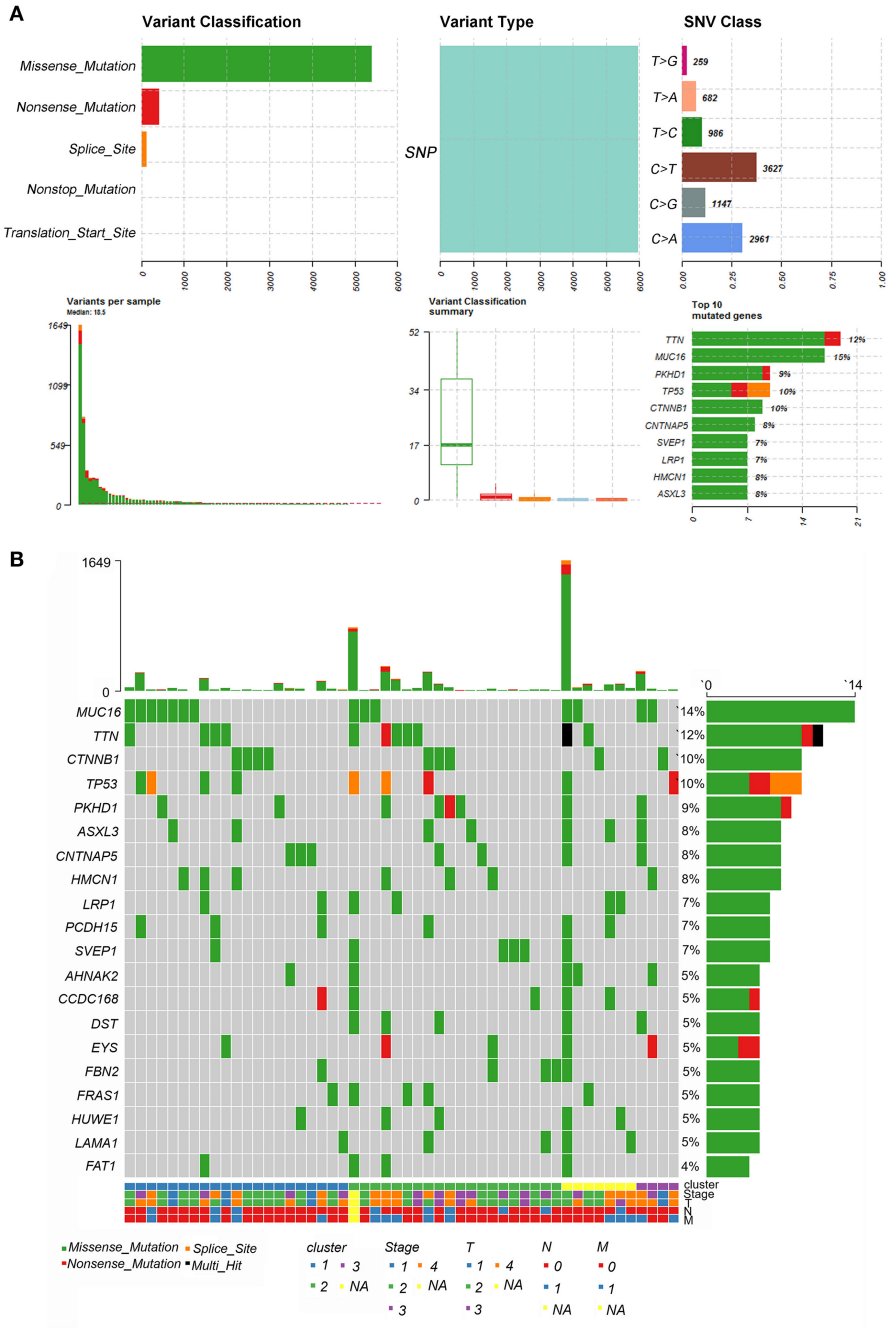
Landscape of TMB in ACC. **(A)** The summary plot of genetic alteration in ACC. **(B)** The oncoplot of genetic alteration in ACC.

Tumor mutational burden (TMB) was considered a promising indicator and exhibited predictive utility in identifying responders and non-responders to immune checkpoint inhibitors (37, 38). Here, the most frequent variants were missense mutations, followed by nonsense mutations and splice sites. SNP was responsible for most variants, and single nucleotide variants (SNVs) mostly occurred as C > T and C > A. The top ten mutated genes in ACC samples were TTN, MUC16, PKHD1, TP53, CTNNB1, CNTNAP5, SVEP1, LRP1, HMCN1, and ASXL3 (Figure 7A). However, we failed to observe significant differences in these genes among the three clusters and other clinical characteristics (Figure 7B).

To further analyze the relationship between m6A key genes and SNPs, we sorted out all the positive polymorphisms based on the SNP2APA database. Eight polymorphisms from four mRNAs (MID1IP1, CEBPZ, CNPY2, and TIPRL) were considered positive by the Kaplan-Meier survival method (Supplementary Table 2). The results of Pearson's correlation analysis indicated that these mRNAs were significantly correlated with the expression level of key m6A-related mRNAs, especially HNRNPA2B1, LRPPRC, and ELAVL1 (Supplementary Figure 6).

### ceRNA Network Construction Based on the Key Gene

**Figure 8:**
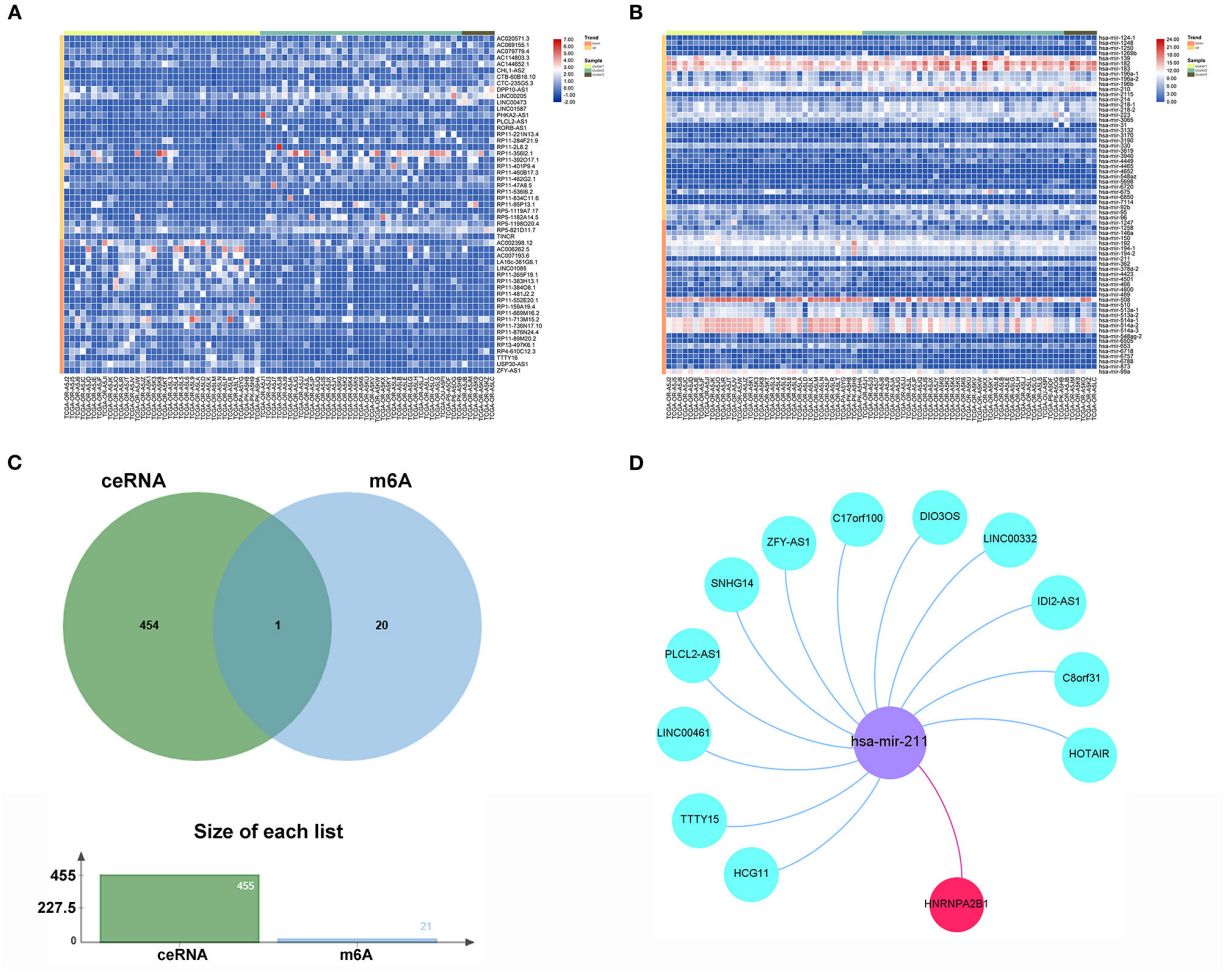
ceRNA network construction of key m6A-related gene. **(A)** The heatmap of significant differential expression lncRNAs. **(B)** The heatmap of significant differential expression miRNAs. **(C)** The Venn plot of 455mRNAs and 21 m6A regulators. **(D)** The ceRNA network of the 12 m6A-related lncRNAs (blue) and target miRNAs (purple) and mRNAs (red).

To reveal the function regulated by the m6A regulator pathway, we obtained 318 lncRNAs and 138 miRNAs in cluster2 compared with cluster1 (Supplementary Tables 3, 4). The heatmap of DEGs with |logFC (fold change)| ≥ 2 and FDR < 0.05 is shown in Figures 8A,B. To further understand how m6A-related lncRNAs mediate mRNA expression by sponging miRNAs, we constructed a ceRNA network based on m6A-related lncRNAs. Twenty-nine lncRNAs were selected from the miRcode database, which targeted 85 miRNAs and 19 miRNAs, were further identified after taking the intersection of DEGs of miRNAs. We then used StarBase to search for mRNAs, and a total of 455 mRNAs were selected based on three databases (miRTarBase, miRDB, and TargetScan) (Supplementary Table 5). Furthermore, taking the intersection of these mRNAs and 21 m6A-related genes as potential functional molecules, these data indicated that 12 lncRNAs (C17orf100, C8orf31, SNHG14, PLCL2-AS1, HCG11, HOTAIR, LINC00332, IDI2-AS1, ZFY-AS1, TTTY15, LINC00461, and DIO3OS) may dysregulate the behavior of hsa-mir-211 so that it promotes the expression of key m6A gene HNRNPA2B1 (Figures 8C,D).

### Pan-Cancer Analysis of the Key Gene

**Figure 9:**
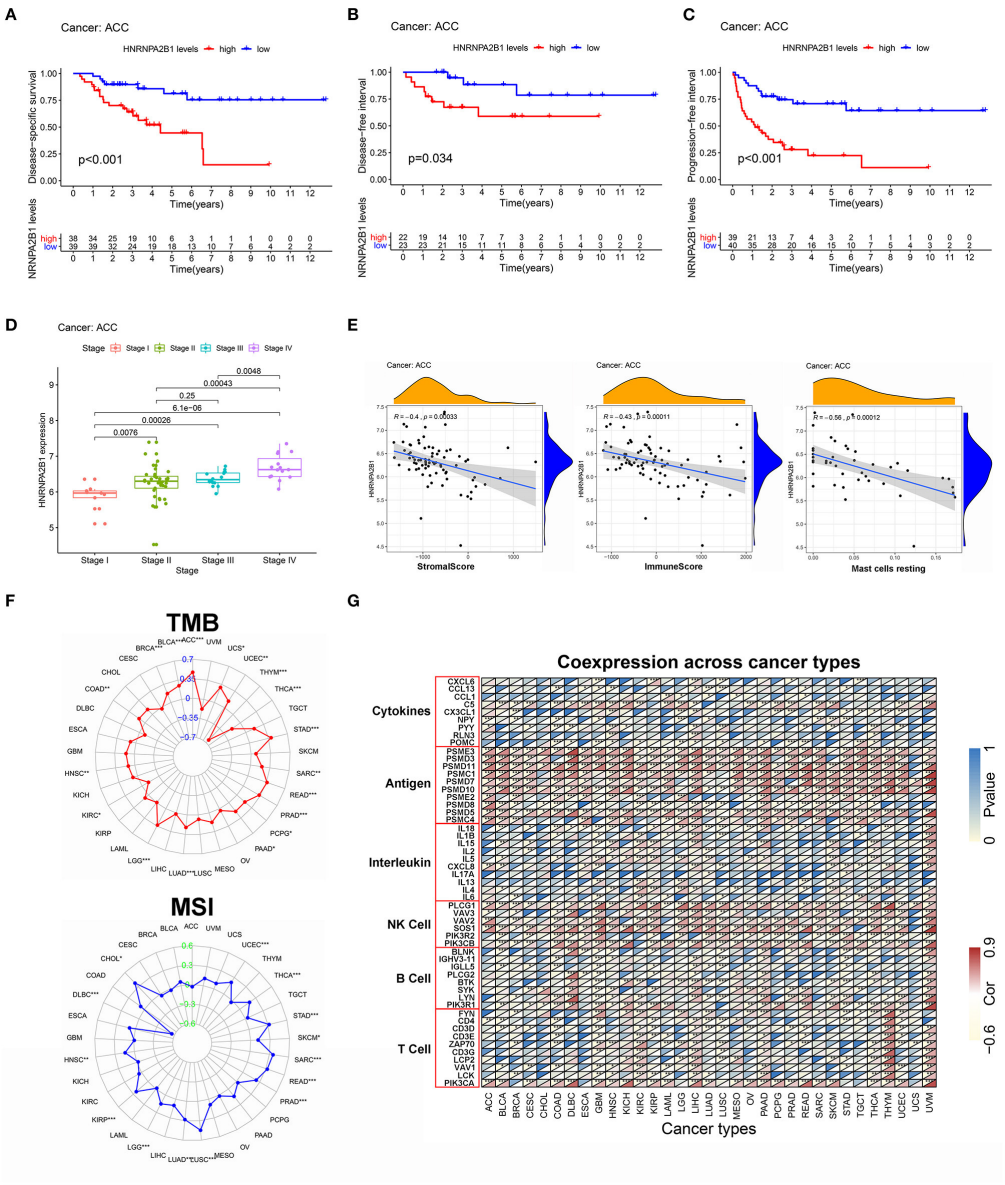
The pan-cancer analysis of HNRNPA2B1. **(A)** The relationship between HNRNPA2B1 expression and DSS. **(B)** The relationship between HNRNPA2B1 expression and DFI. **(C)** The relationship between HNRNPA2B1 expression and PFI. **(D)** The HNRNPA2B1 expression was significantly correlated with TNM stage. **(E)** The correlation among HNRNPA2B1 expression and immune landscape. **(F)** Pan-cancer analysis of the relationship between HNRNPA2B1 expression and TMB (above) or MSI (below). **(G)** The correlation among HNRNPA2B1 expression and immune-related mRNAs.

To further confirm the key role of HNRNPA2B1 in the m6A modification process, we adopted a pan-cancer analysis and downloaded all the data from the UCSC Cancer Genomics Browser (https://genome-cancer.ucsc.edu), which offers interactive visualization and exploration of TCGA genomic and clinical data. HNRNPA2B1 expression was re-evaluated and was notably found to significantly impact prognosis in ACC. High HNRNPA2B1 expression was associated with decreased disease-specific survival (DSS) (*P* < 0.001), disease-free interval (DFI) (*P* = 0.034), and progression-free interval (PFI) (*P* < 0.001) (Figures 9A–C). In addition, as shown in Figure 9D, the expression of HNRNPA2B1 was significantly correlated with TNM stage. Furthermore, we analyzed the correlation among HNRNPA2B1 expression, ESTIMATE score, and infiltrating immune cells. The results showed that HNRNPA2B1 levels were significantly negatively correlated with immune score (*r* = −0.43, *P* = 0.00011), stromal score (*r* = −0.40, *P* = 0.00033), and resting mast cells (*r* = −0.56, *P* = 0.00012) (Figure 9E). Additionally, increased TMB has been linked to PD1/PD-L1 therapeutic response, and we found that HNRNPA2B1 mRNA levels were correlated to multiple types of cancers, including ACC, stomach adenocarcinoma (STAD), thymoma (THYM) etc. Microsatellite instability (MSI) is a pattern of hypermutation caused by defects in the mismatch repair system and has been known to be both predictive and prognostic to better profile responses to anti-PD-1 immunotherapy (39). Next, we found that HNRNPA2B1 was associated with diffuse large B-cell lymphoma (DBLC) and lung squamous cell carcinoma (LUSC), but not ACC, as shown in the radar plot (Figure 9F). Moreover, Spearman's correlation was used to show the relationship between HNRNPA2B1 and immune-related mRNAs in 33 cancer types (Figure 9G).

## Discussion

With a heterogeneous clinical characteristic and poor OS, treating this complex malignant tumor, adrenocortical carcinoma, is a substantial clinical challenge (40). Further, the current TNM classification system remains inapplicable to predict prognosis. Here, we used univariate and multivariate analyses to evaluate the clinical prognostic value, and the results indicated that most clinical characteristics, including M stage and N stage, failed to guide treatment options. Previous studies have shown that the invasion and proliferation of ACC is regulated by genomic molecular characteristics (41). m6A is the most common and plentiful modification to affect cancer development through the regulation of m6A methyltransferases, demethylases, and binding proteins, but to date, the potential role of m6A regulators in ACC prognosis is not well-understood. Based on the TCGA dataset, we identified three clusters according to the optimal k-means clustering, and we observed a significant difference in OS among the three clusters, suggesting that the expression of m6A-related regulators is intimately related to the prognosis and malignancy of ACC.

Accumulating studies have focused on the tumor immune microenvironment and ACC is characterized as a highly immunogenic tumor, with 86.3% of ACC specimens showing high rates of tumor infiltrating lymphocytes (TILs) (42). Here, GO, KEGG, and GSEA analyses revealed that the DEGs among clusters were enriched in immune-related signaling pathways, such as IL-17 signaling pathway. Furthermore, the ESTIMATE score, especially the immune score, was significantly correlated with the expression of m6A patterns. Similarly, the infiltration of immune cells (M1, M2 macrophages, and NK cells activated) was significantly increased and the infiltration of immune cells (dendritic cells, macrophages M0, and NK cells activated) was significantly decreased in the low-risk score group (cluster1) compared with the high-risk score groups (cluster2 or cluster3). These results indicate a comprehensive evaluation of the m6A modification patterns that will facilitate understanding the characteristics of TME cell infiltration and promote individualized novel therapies by determining the response to immunotherapy.

Here, we systematically explored the effects of multiple m6A regulators on OS in ACC and attempted to construct a risk model for prediction. Next, we distinguished a prognostic risk signature with eight identified m6A regulators (METTL14, ZC3H13, FTO, YTHDF1, YTHDF3, HNRNPA2B1, LRPPRC, and ELAVL1), which divided the OS in ACC into high-risk subgroups with high mortality and low-risk subgroup with remarkably better survival. Notably, compared with the previous prognostic markers (T, N, M clinical stage), our prognostic risk signature can achieve higher accuracy, with AUC values >0.8. In summary, the risk signature we constructed might be viewed as a new potential and promising biomarker that can provide more precise clinical applications and an efficient guide for treatment. However, owing to the limited number of samples in most GEO databases, this model needs to be re-confirmed in other databases with large populations. Meanwhile, we applied survival analysis to further detect the eight identified genes in the TCGA and GEO datasets, and we served overlapping m6A regulators as significant biomarkers, particularly HNRNPA2B1, which was proven to have a positive association with poor OS, EFS, DSS, DFI, and PFI in TCGA and all GEO datasets.

The role of HNRNPA2B1 in cancer has recently garnered increasing attention. On the one hand, HNRNPA2B1 acted as a nuclear m6A reader that recruited the miRNA microprocessor complex protein DGCR8 to a subset of precursor miRNAs and mediated the mature miRNA processing (43). On the other hand, HNRNPA2B1 functioned as an adaptor and modulated the molecular changes to alternative splicing combined with METTL3 (44). Here, the results showed that lower immune scores, stromal scores, and ESTIMATE scores were significantly associated with higher HNRNPA2B1 expression, which demonstrated that this m6A gene signature played a non-negligible role in shaping diverse stromal and immune TME landscapes, implying that HNRNPA2B1 may affect the therapeutic efficacy of immune checkpoint blockade. Moreover, using the CIBERSORT algorithm, we found that HNRNPA2B1 mediated the TME infiltration patterns to accelerate ACC progression partly by regulating macrophages M0. Tumor mutational burden, defined as the total number of somatic coding mutations per million bases, has emerged as a notable biomarker of response to immunotherapy (45). Recent studies showed that HNRNPA2B1 with high frequency of mutation may have an influence on promoting tumorigenesis in melanoma (46). Similarly, by analyzing the mutation annotation files of the TCGA cohort, the results showed that HNRNPA2B1 has a close correlation with TMB in multiple types of cancer, particularly ACC. Based on the SNP2APA database, we identified four highly variant mutated genes, most of which were highly correlated with the HNRNPA2B1 expression level. These findings indicated that high HNRNPA2B1 expression is related to dysregulation of the TME landscape and a sharp accumulation of gene mutations, thus becoming a promising therapeutic target for ACC.

Furthermore, several selected lncRNAs were reported to be associated with cancer progression (47), but there have been few reports on lncRNAs regarding ACC progression, and how m6A-related genes act in an lncRNA-miRNA-dependent manner during ACC progression is still unknown. In our research, we constructed a ceRNA network to target m6A-related miRNA and lncRNAs, which consist of twelve lncRNAs and one miRNA. Therefore, we should pay more attention to filter key lncRNAs that could predict OS and EFS and have a close relationship with HNRNPA2B1. Finally, we identified two lncRNAs, HOTAIR and IDI2-AS1, according to the above-mentioned conditions. Located on chromosome.12q13.13, lncRNA HOTAIR (HOX Transcript Antisense Intergenic RNA) was regarded as a regulator of chromatin states (48). In immune cells, HOTAIR has the ability to interfere with the TME landscape by inducing IκBα degradation, with the consequent activation of NF-κB pathways and secretion of pro-inflammatory cytokines (49, 50). Moreover, HOTAIR also led to the downregulation of the tumor suppressor gene SETD2, promoting MSI and high TMB (50, 51). In ACC, HOTAIR is overexpressed in tumor tissues compared with normal tissues. For *in vitro* experiments, HOTAIR can prompt the progression of ACC by shortening the cell cycle and promoting the proliferation of ACC cells (52). Hence, we planned to take the next step to deeply explore the interaction of HOTAIR and HNRNPA2B1 *in vitro*. In our study, IDI2-AS1 was also found to play a significant role in ACC. However, there are few reports on how IDI2-AS1 acts as an oncogene. Thus, we carried out a pan-cancer analysis of IDI2-AS1 and hoped our results help to identify the prognostic value. We found that high IDI2-AS1 expression was obviously associated with poor DSS, DFI, PFI, and high TMB rate. Furthermore, the findings revealed that IDI2-AS1 had a stable association with the expression of PD1 (PDCD1) and PD-L1 (CD274) to serve as a potential prognostic marker or therapeutic targets of cancers integrated with m6A-related regulators (Supplementary Figure 7). Moreover, previous studies have reported the different functions of miR-211, which can facilitate platinum chemosensitivity by blocking the DNA damage response (53) or by stimulating the emergence of BRAF inhibitor resistance (54). Most speculations that miR-211 may regulate the m6A modification pathway need to be confirmed by further validation or experiments.

In conclusion, this study is the first to comprehensively identify and systematically profile the gene signatures of m6A-related regulators in ACC. The different m6A modification patterns played an important role in the heterogeneity and complexity of the TME. We also developed an eight-gene-signature prognostic model, in particular HNRNPA2B1, which might determine the clinical progression of ACC. Moreover, we constructed a ceRNA network to further decipher the molecular mechanisms based on HNRNPA2B1. Our results indicate that m6A genes are promising predictive indicators that may provide novel insights into ACC therapeutic strategies and guide effective immunotherapy.

## Data Availability Statement

The datasets presented in this study can be found in online repositories. The names of the repository/repositories and accession number(s) can be found in the article/Supplementary Material.

## Ethics Statement

Written informed consent was obtained from the individual(s), and minor(s)' legal guardian/next of kin, for the publication of any potentially identifiable images or data included in this article.

## Author Contributions

KC: designed the study. YJ: analyzed, interpreted the data, and wrote original draft. ZW, DH, YZ, and XH: wrote this manuscript. LG, MX, XC, and YC: edited and revised the manuscript. All authors have seen and approved the final version of the manuscript.

## Conflict of Interest

The authors declare that the research was conducted in the absence of any commercial or financial relationships that could be construed as a potential conflict of interest.

## Supplementary Material

Supplementary Figure 1 Consensus clustering matrix for k = 2 to 5.

Supplementary Figure 2 **(A)** The univariate analysis of age, sex, M, N, T, and clinic stage. **(B)** The multivariate analysis of age, sex, M, N, T, and clinic stage.

Supplementary Figure 3 Prognostic value of risk model, YTHDF1 and YTHDF3. **(A)** Kaplan-Meier OS curves and EFS curves for patients in risk model. **(B)** Kaplan-Meier OS curves and EFS curves for patients in YTHDF1. **(C)** Kaplan-Meier OS curves and EFS curves for patients in YTHDF3.

Supplementary Figure 4 Prognostic value of FTO, METTL14 and ZC3H13. **(A)** Kaplan-Meier OS curves and EFS curves for patients in FTO. **(B)** Kaplan-Meier OS curves and EFS curves for patients in METTL14. **(C)** Kaplan-Meier OS curves and EFS curves for patients in ZC3H13.

Supplementary Figure 5 Verification of key m6A regulators expression in immunohistochemistry. **(A)** The expression of HNRNPA2B1 in tumor and normal tissue. **(B)** The expression of LRPPRC in tumor and normal tissue. **(C)** The expression of ELAVL1 in tumor and normal tissue. (***P < 0.001; **P < 0.01).

Supplementary Figure 6 Pearson's correlation analysis of positive polymorphisms and key m6A regulators.

Supplementary Figure 7 The pan-cancer analysis of IDI2-AS1. **(A)** The relationship between IDI2-AS1 expression and OS. **(B)** The relationship between IDI2-AS1 expression and DSS. **(C)** The relationship between IDI2-AS1 expression and DFI. **(D)** The relationship between IDI2-AS1 expression and PFI. **(E)** The correlation among IDI2-AS1 expression and immune landscape. **(F)** Pan-cancer analysis of the relationship between IDI2-AS1 expression and TMB. **(G)** The correlation among IDI2-AS1 expression and PD1/PD-L1 from starBase V3.0 project.

Supplementary Table 1 Cox analysis of 21 m6A genes in ACC patients.

Supplementary Table 2 Positive polymorphisms based on SNP2APA database.

Supplementary Table 3 All differential expression lncRNAs in cluster2 compared with cluster1.

Supplementary Table 4 All differential expression miRNAs in cluster2 compared with cluster1.

Supplementary Table 5 Targeted mRNAs base on miRTarBase, miRDB, and TargetScan databases.

## Abbreviations

ACC: adrenocortical carcinoma
TNM: tumor, lymph node, and metastasis
m6A: N6-methyladenosine
TME: the tumor microenvironment
BMDCs: bone marrow-derived cells
TCGA: the cancer genome atlas
GEO: the gene expression omnibus database
GDC: genomic data commons
OS: the overall survival
DEG: differentially expressed gene
GSEA: gene set enrichment analysis
LASSO: the least absolute shrinkage and selection operator
HR: the hazard ratio
ROC: the receiver operating characteristic
SNP: single nucleotide polymorphism
AUC: the area under the curve
EFS: the event-free survival
TMB: the tumor burden
SNV: single nucleotide variants
DDS: the disease-specific survival
DFI: the disease-free interval
PFI: the progression-free interval
TILs: tumor infiltrating lymphocytes
HOTAIR: HOX transcript antisense intergenic RNA.
