# Supplementary figures and images for: Analysis of m6A-Related Signatures in the Tumor Immune Microenvironment and Identification of Clinical Prognostic Regulators in Adrenocortical Carcinoma
Source: Front Immunol. 2021 Mar 3;12:637933. doi: 10.3389/fimmu.2021.637933 (PMC7966528; doi:10.3389/fimmu.2021.637933)

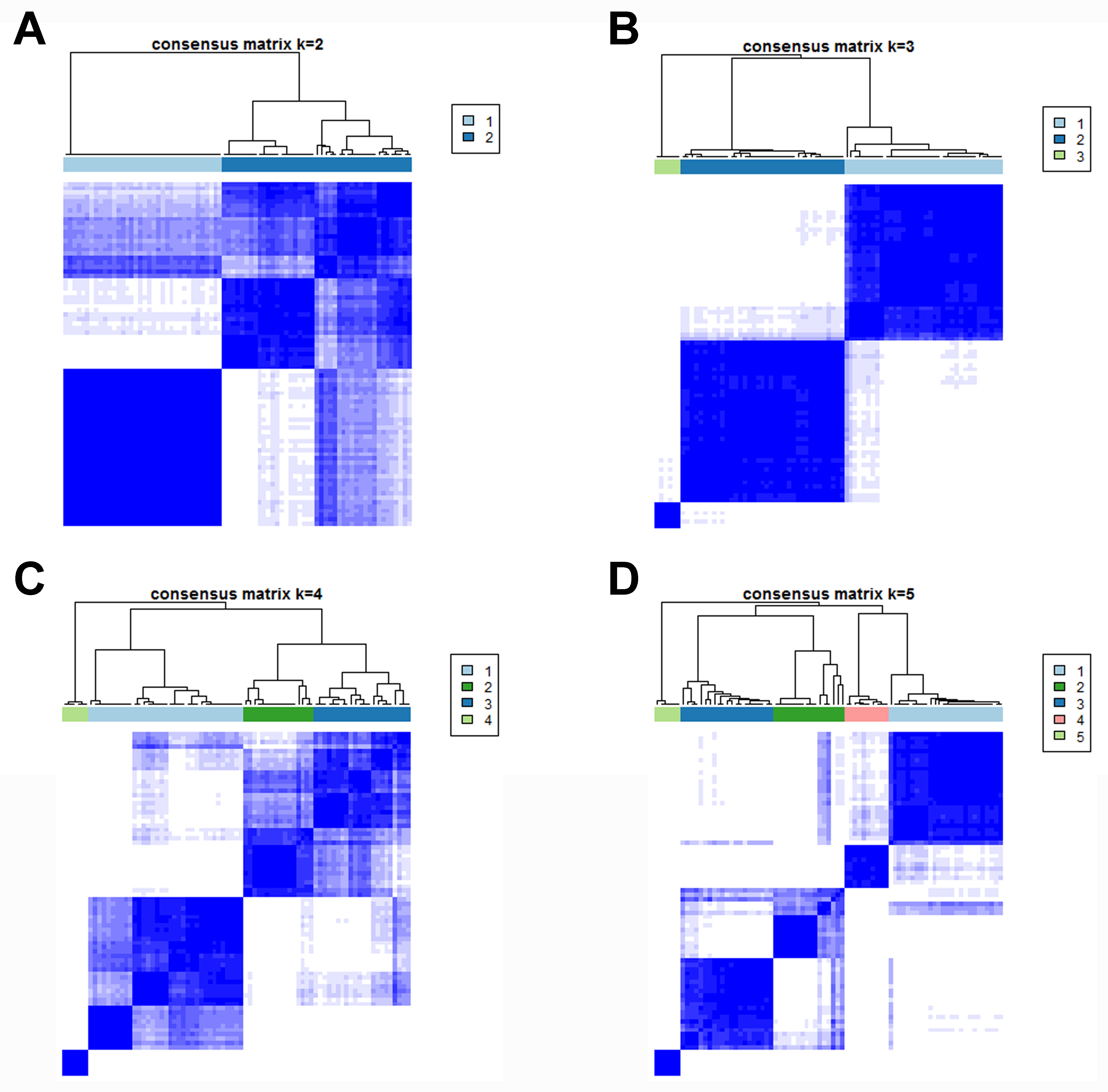

Supplement: Supplementary Figure 1 — Consensus clustering matrix for k = 2 to 5. [file Image_1.TIF]

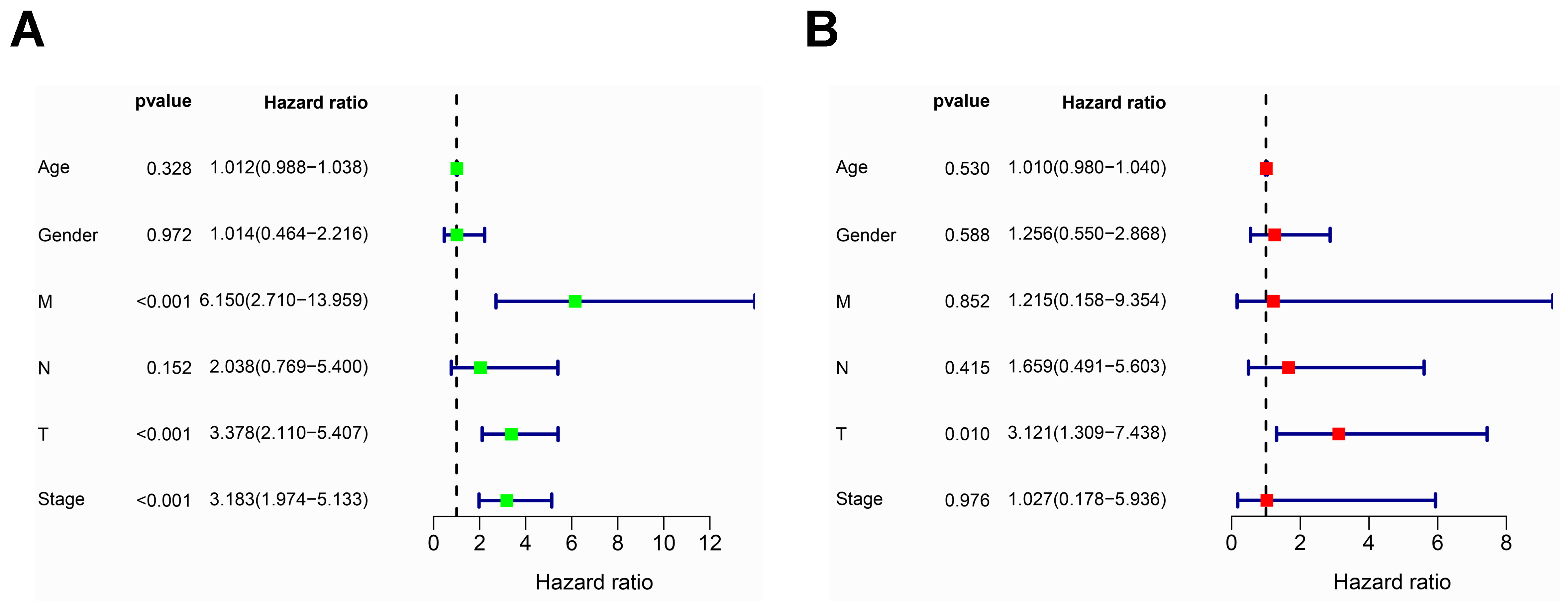

Supplement: Supplementary Figure 2 — (A) The univariate analysis of age, sex, M, N, T, and clinic stage. (B) The multivariate analysis of age, sex, M, N, T, and clinic stage. [file Image_2.TIF]

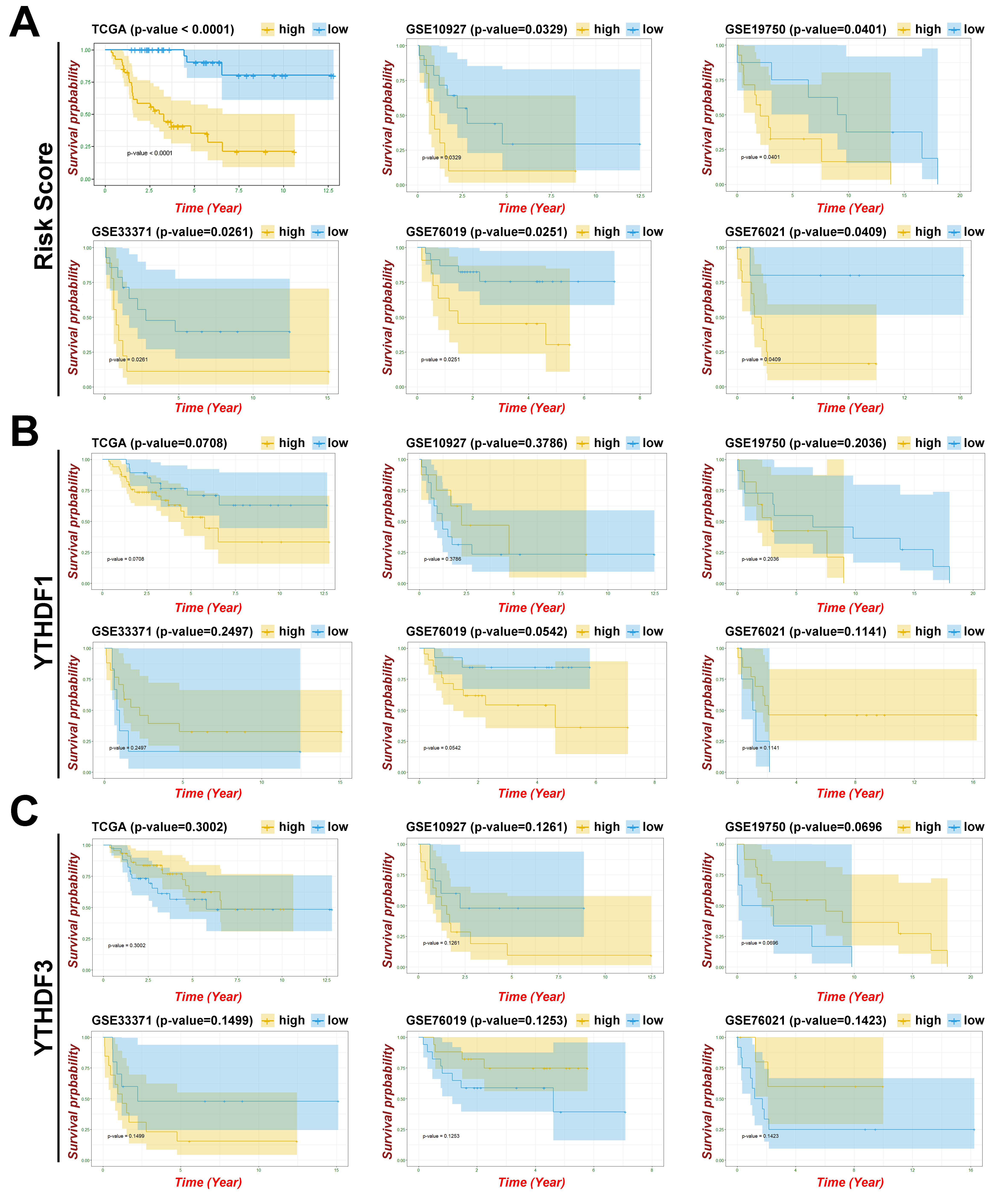

Supplement: Supplementary Figure 3 — Prognostic value of risk model, YTHDF1 and YTHDF3. (A) Kaplan-Meier OS curves and EFS curves for patients in risk model. (B) Kaplan-Meier OS curves and EFS curves for patients in YTHDF1. (C) Kaplan-Meier OS curves and EFS curves for patients in YTHDF3. [file Image_3.TIF]

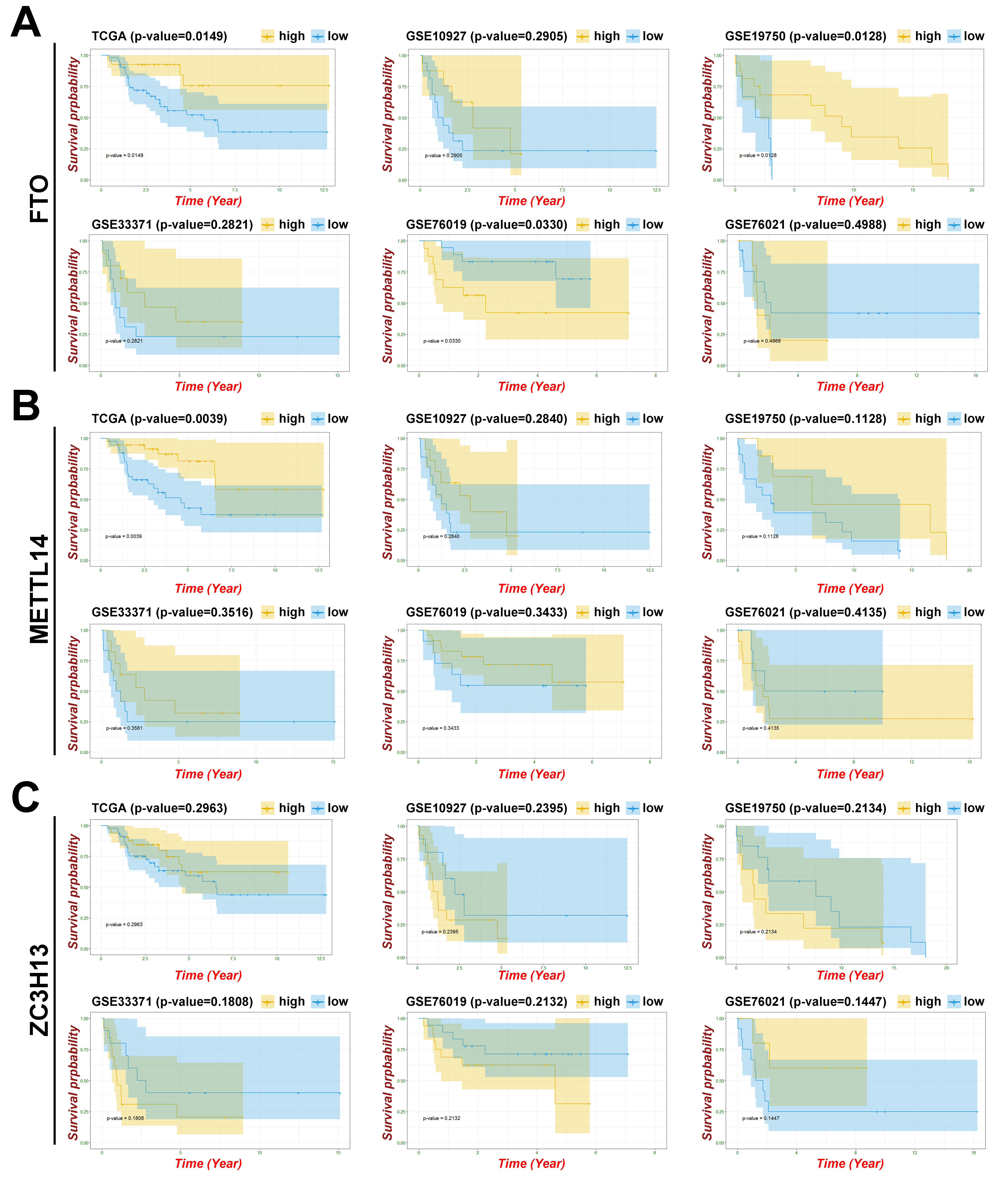

Supplement: Supplementary Figure 4 — Prognostic value of FTO, METTL14 and ZC3H13. (A) Kaplan-Meier OS curves and EFS curves for patients in FTO. (B) Kaplan-Meier OS curves and EFS curves for patients in METTL14. (C) Kaplan-Meier OS curves and EFS curves for patients in ZC3H13. [file Image_4.TIF]

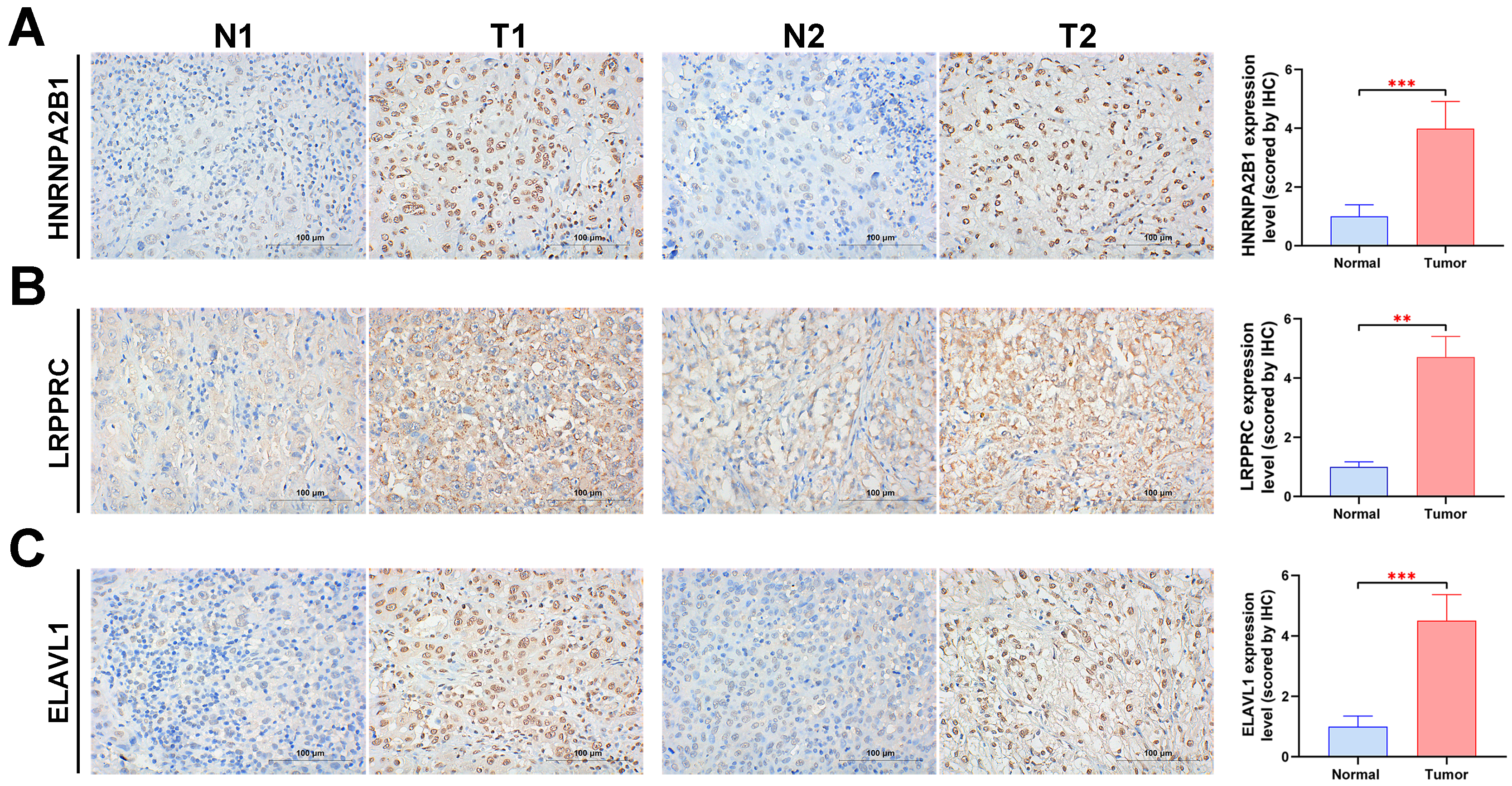

Supplement: Supplementary Figure 5 — Verification of key m6A regulators expression in immunohistochemistry. (A) The expression of HNRNPA2B1 in tumor and normal tissue. (B) The expression of LRPPRC in tumor and normal tissue. (C) The expression of ELAVL1 in tumor and normal tissue. (***P < 0.001; **P < 0.01). [file Image_5.tif]

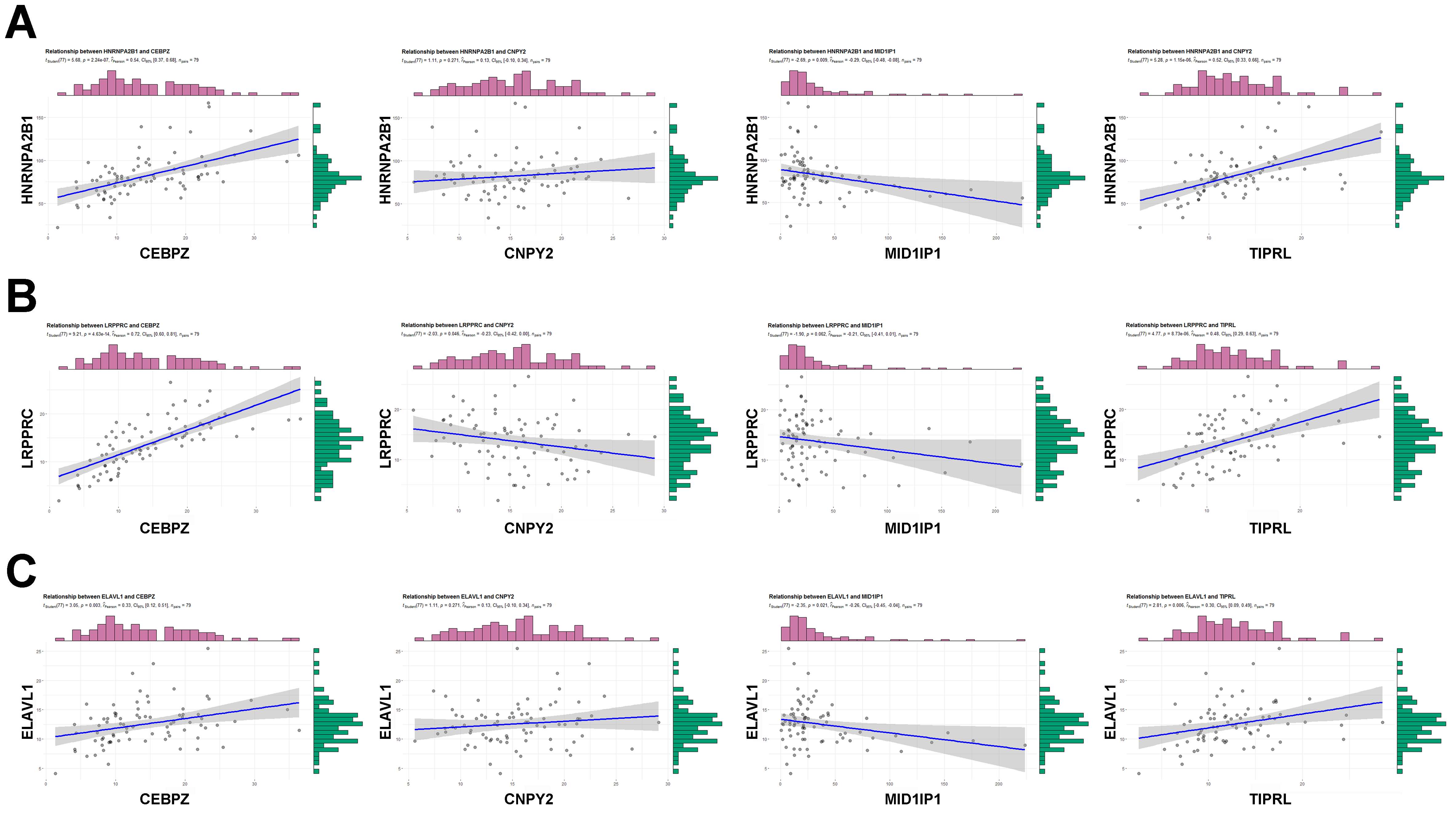

Supplement: Supplementary Figure 6 — Pearson's correlation analysis of positive polymorphisms and key m6A regulators. [file Image_6.TIF]

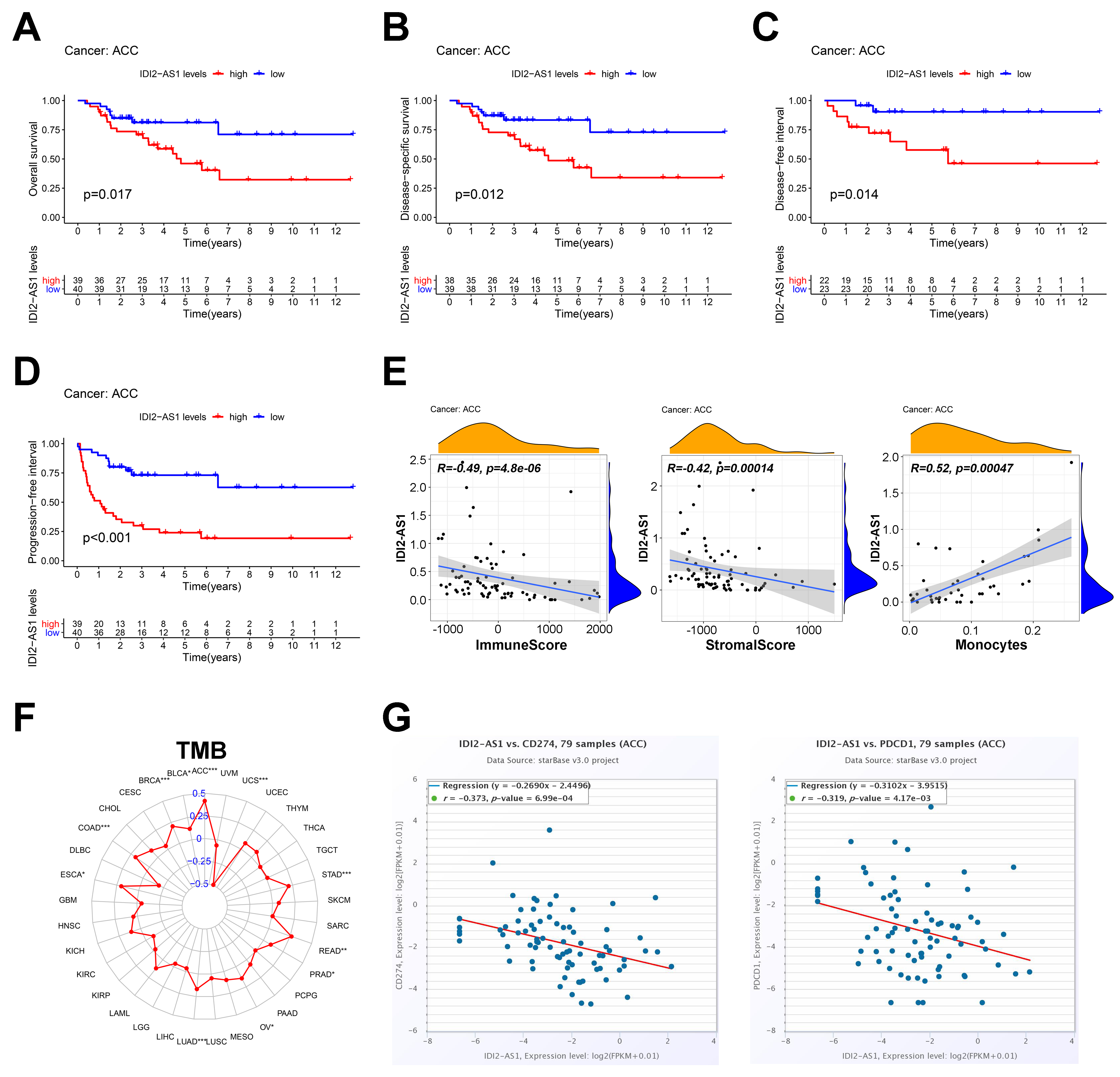

Supplement: Supplementary Figure 7 — The pan-cancer analysis of IDI2-AS1. (A) The relationship between IDI2-AS1 expression and OS. (B) The relationship between IDI2-AS1 expression and DSS. (C) The relationship between IDI2-AS1 expression and DFI. (D) The relationship between IDI2-AS1 expression and PFI. (E) The correlation among IDI2-AS1 expression and immune landscape. (F) Pan-cancer analysis of the relationship between IDI2-AS1 expression and TMB. (G) The correlation among IDI2-AS1 expression and PD1/PD-L1 from starBase V3.0 project. [file Image_7.TIF]
