# Supplementary material for: Analysis of m6A-Related Signatures in the Tumor Immune Microenvironment and Identification of Clinical Prognostic Regulators in Adrenocortical Carcinoma
Source: Front Immunol. 2021 Mar 3;12:637933. doi: 10.3389/fimmu.2021.637933 (PMC7966528; doi:10.3389/fimmu.2021.637933)
Supplement: Supplementary Table 1 — Cox analysis of 21 m6A genes in ACC patients. [file Data_Sheet_1.docx]

**Supplementary Table S1**

**Cox analysis of 21 m6A genes in ACC patients**

| id | HR | HR.95L | HR.95H | pvalue |
| --- | --- | --- | --- | --- |
| YTHDF2 | 1.088492 | 1.00906 | 1.174177 | 0.028289 |
| RBM15 | 2.002019 | 1.408489 | 2.845659 | 0.000109 |
| LRPPRC | 1.101648 | 1.022193 | 1.187279 | 0.011254 |
| RBM15B | 1.156506 | 0.995661 | 1.343334 | 0.057034 |
| YTHDC1 | 1.052897 | 0.911968 | 1.215604 | 0.482015 |
| METTL14 | 0.804862 | 0.632848 | 1.023632 | 0.076799 |
| YTHDC2 | 0.930417 | 0.758784 | 1.140871 | 0.488171 |
| WTAP | 1.078088 | 0.995141 | 1.167948 | 0.065662 |
| HNRNPA2B1 | 1.047604 | 1.032201 | 1.063237 | 7.57E-10 |
| CBLL1 | 1.163332 | 0.957122 | 1.41397 | 0.128579 |
| YTHDF3 | 0.980387 | 0.902248 | 1.065292 | 0.640195 |
| KIAA1429 | 1.171334 | 0.952117 | 1.441024 | 0.134694 |
| ZC3H13 | 0.99237 | 0.795841 | 1.237431 | 0.945768 |
| HNRNPC | 1.044613 | 1.019071 | 1.070796 | 0.000549 |
| METTL3 | 1.095668 | 0.947357 | 1.267196 | 0.218245 |
| FTO | 0.859178 | 0.727172 | 1.015147 | 0.074534 |
| ALKBH5 | 1.002789 | 0.97755 | 1.028679 | 0.830443 |
| IGF2BP1 | 1.103706 | 1.016284 | 1.198649 | 0.019098 |
| ELAVL1 | 1.211508 | 1.095701 | 1.339554 | 0.000182 |
| YTHDF1 | 1.037607 | 0.978382 | 1.100417 | 0.218271 |
| FMR1 | 0.936704 | 0.782724 | 1.120976 | 0.475463 |

**Supplementary Table S2**

**Positive polymorphisms based on SNP2APA database**

| SNP ID | mRNA | SNP position | Alleles | KM P value |
| --- | --- | --- | --- | --- |
| rs41392645 | CNPY2 | chr12:56696742 | A/G | 0.00000257 |
| rs56998789 | TIPRL | chr1:168119771 | G/A | 0.00014 |
| rs73030238 | TIPRL | chr1:168111188 | C/A | 0.00014 |
| rs12466414 | MID1IP1 | chr2:227022119 | C/T | 3.51e-11 |
| rs12485003 | CEBPZ | chr22:40635276 | C/T | 0.0000245 |
| rs16866885 | MID1IP1 | chr2:227078807 | G/A | 0.0000395 |
| rs2396304 | MID1IP1 | chr2:227074596 | T/C | 0.0000395 |
| rs77904070 | MID1IP1 | chr2:227067213 | G/C | 0.0000395 |

**Supplementary Table S3**

**All differential expression lncRNAs in the cluster2 compared to cluster1**

| lncRNAs | logFC | logCPM | PValue |
| --- | --- | --- | --- |
| RP11-265F19.1 | -4.553 | 9.759639 | 3.67E-12 |
| PHKA2-AS1 | 3.363454 | 10.32668 | 2.96E-10 |
| RP11-713M15.2 | -2.83914 | 11.34706 | 2.37E-09 |
| AC006262.5 | -4.35539 | 12.32056 | 6.97E-09 |
| TTTY16 | -4.40672 | 9.231285 | 2.85E-08 |
| RP1-159A19.4 | -2.38697 | 9.446272 | 3.04E-08 |
| USP30-AS1 | -1.56548 | 9.573988 | 5.55E-08 |
| RP13-497K6.1 | -3.2117 | 9.46804 | 8.27E-08 |
| AC069155.1 | 2.64759 | 9.492803 | 9.11E-08 |
| PLCL2-AS1 | 3.927634 | 9.202045 | 1.68E-07 |
| AC002398.12 | -2.08561 | 10.23671 | 2.52E-07 |
| RP11-736N17.10 | -2.74511 | 9.100848 | 4.07E-07 |
| LA16c-381G6.1 | -2.88025 | 9.466042 | 4.09E-07 |
| AC007193.6 | -3.04736 | 10.27774 | 4.91E-07 |
| LINC00205 | 1.182585 | 10.48284 | 6.47E-07 |
| DPP10-AS1 | 2.093567 | 10.25406 | 6.62E-07 |
| RP11-47A8.5 | 2.671609 | 10.11119 | 7.94E-07 |
| RP11-834C11.6 | 5.409664 | 10.74694 | 9.71E-07 |
| CHL1-AS2 | 3.024207 | 8.999243 | 4.09E-06 |
| RP5-1198O20.4 | 1.371811 | 9.411247 | 5.58E-06 |
| RP11-462G2.1 | 2.152331 | 9.715787 | 5.66E-06 |
| RP5-821D11.7 | 1.126985 | 10.35362 | 5.73E-06 |
| RP4-610C12.3 | -1.92433 | 9.151913 | 6.84E-06 |
| RP5-1182A14.5 | 2.835254 | 10.55676 | 1.21E-05 |
| AC079779.4 | 1.46228 | 9.578687 | 1.22E-05 |
| RP11-2L8.2 | 3.848532 | 10.16232 | 1.71E-05 |
| RP11-284F21.9 | 1.977933 | 9.621456 | 2.09E-05 |
| ZFY-AS1 | -2.66393 | 8.840352 | 2.84E-05 |
| RP11-401P9.4 | 2.619971 | 10.11612 | 4.14E-05 |
| LINC01085 | -2.15464 | 9.936421 | 5.02E-05 |
| RP11-669M16.2 | -1.88656 | 8.895331 | 5.64E-05 |
| AC020571.3 | 2.492403 | 9.085782 | 6.13E-05 |
| AC144652.1 | 1.232629 | 9.984538 | 6.47E-05 |
| RP11-481J2.2 | -1.84695 | 8.997229 | 6.84E-05 |
| RP11-383H13.1 | -1.92057 | 9.311215 | 6.93E-05 |
| RP11-460B17.3 | 1.72589 | 9.055024 | 7.40E-05 |
| CTB-60B18.10 | 2.166758 | 8.887774 | 8.60E-05 |
| LINC01587 | 2.274853 | 9.127111 | 9.78E-05 |
| RP11-89M20.2 | -2.57631 | 8.923304 | 0.000102 |
| RP11-356I2.1 | 2.679349 | 12.89532 | 0.000102 |
| RP11-552E20.1 | -3.15728 | 8.91126 | 0.000105 |
| RP11-876N24.4 | -1.38566 | 9.355343 | 0.000123 |
| RP11-392O17.1 | 2.60034 | 10.75849 | 0.000125 |
| RP11-95P13.1 | 2.774536 | 11.58269 | 0.000139 |
| RP11-384O8.1 | -1.48497 | 9.344133 | 0.00014 |
| RORB-AS1 | 2.067411 | 8.968805 | 0.000156 |
| AC114803.3 | 1.810847 | 9.20647 | 0.000157 |
| LINC00473 | 1.888248 | 9.329025 | 0.000158 |
| TINCR | 1.749189 | 8.928182 | 0.000187 |
| CTC-235G5.3 | 2.051167 | 9.148467 | 0.000188 |
| RP5-1119A7.17 | 2.173165 | 8.856928 | 0.0002 |
| RP11-221N13.4 | 2.541995 | 8.946499 | 0.000212 |
| RP11-536I6.2 | 3.050305 | 8.95601 | 0.000212 |
| AC005355.2 | -1.65231 | 8.983017 | 0.000265 |
| PRKCQ-AS1 | 1.72877 | 8.911811 | 0.000268 |
| RP11-114G11.5 | 1.918718 | 8.960661 | 0.000271 |
| RP11-554A11.9 | 2.424518 | 8.915515 | 0.000282 |
| LINC00887 | -1.31395 | 9.481542 | 0.000292 |
| RP11-1246C19.1 | 1.177215 | 9.198595 | 0.000297 |
| RP11-118B22.4 | -1.79262 | 8.927172 | 0.00031 |
| CTD-3138F19.1 | -2.996 | 9.2759 | 0.000317 |
| RP11-479O16.1 | 2.460076 | 9.069195 | 0.000318 |
| PWAR6 | 1.148121 | 9.293528 | 0.000327 |
| RP11-10A14.5 | 2.329185 | 8.991705 | 0.000348 |
| HOXA11-AS | 1.433523 | 10.12077 | 0.00035 |
| RP11-881M11.4 | -1.62215 | 9.491806 | 0.000375 |
| RP11-848P1.3 | -1.10453 | 9.573709 | 0.000391 |
| CTD-3007L5.1 | -2.65978 | 8.844237 | 0.000407 |
| RP11-54O7.3 | 1.406096 | 9.160957 | 0.000413 |
| RP11-678G14.3 | -2.31188 | 11.0996 | 0.000464 |
| RP11-54O7.16 | 1.61072 | 9.395528 | 0.000471 |
| AC006538.1 | 1.466636 | 9.124361 | 0.000501 |
| DGCR9 | 1.403043 | 8.950953 | 0.000529 |
| RP11-390F4.3 | 1.544876 | 9.026823 | 0.000531 |
| RP11-167B3.3 | -2.43383 | 9.062009 | 0.000609 |
| RP4-549F15.1 | 1.735953 | 9.070899 | 0.000612 |
| RP11-738B7.1 | 1.700153 | 8.822902 | 0.000613 |
| LINC00880 | -2.35223 | 8.840375 | 0.000653 |
| RP11-163F15.1 | -2.53364 | 8.832089 | 0.000653 |
| RP11-284H18.1 | 1.468145 | 9.250854 | 0.000676 |
| LINC01314 | 2.074749 | 9.534159 | 0.000686 |
| SNHG14 | 1.101811 | 9.253359 | 0.000694 |
| C8orf31 | -1.12439 | 10.2701 | 0.000707 |
| RP11-62H20.1 | 1.434261 | 9.496003 | 0.000723 |
| AC007091.1 | -1.79664 | 8.918125 | 0.00073 |
| CTA-384D8.31 | -2.24026 | 9.004169 | 0.000743 |
| PCAT7 | 1.75813 | 9.115519 | 0.000805 |
| RP11-90K6.1 | -1.98167 | 9.23144 | 0.000846 |
| HCP5 | -1.2346 | 11.24176 | 0.000965 |
| MIR503HG | 1.068199 | 11.84071 | 0.000985 |
| AC116614.1 | -1.44482 | 9.768268 | 0.001114 |
| COLCA1 | -1.41009 | 8.86756 | 0.001137 |
| HOXC13-AS | 1.72351 | 8.970106 | 0.00115 |
| RP11-115N4.1 | 1.932479 | 8.82135 | 0.001153 |
| RP11-231N3.1 | -1.9999 | 8.751681 | 0.001169 |
| CTD-3128G10.7 | -1.51562 | 8.8804 | 0.001195 |
| RP11-459I19.1 | -1.22559 | 9.05339 | 0.001228 |
| LINC00839 | 1.709684 | 9.407676 | 0.001231 |
| LINC01432 | -2.45642 | 8.811005 | 0.001263 |
| RP3-395M20.8 | -1.22885 | 9.400087 | 0.001301 |
| MIR210HG | 1.101546 | 10.3951 | 0.001349 |
| RP11-888D10.3 | 1.307092 | 9.013464 | 0.001352 |
| RP1-86C11.7 | 1.171321 | 9.159732 | 0.001353 |
| RP11-380P13.1 | -1.97893 | 8.785908 | 0.001379 |
| CTA-14H9.5 | -1.10361 | 9.665281 | 0.001388 |
| LINC00460 | 2.034183 | 8.791205 | 0.001441 |
| RP11-546K22.1 | -1.89999 | 8.840099 | 0.001522 |
| RP11-284F21.10 | 1.310515 | 10.74458 | 0.001524 |
| RP11-791G15.2 | 1.005716 | 9.902114 | 0.001557 |
| RP11-326C3.7 | 1.012606 | 9.369706 | 0.001595 |
| CTB-31N19.3 | 1.481224 | 10.07968 | 0.001669 |
| RP11-493L12.4 | -1.08498 | 9.124454 | 0.001759 |
| RP4-601P9.2 | 1.205272 | 9.077245 | 0.001779 |
| LINC01476 | 2.348912 | 8.798383 | 0.001995 |
| LINC00511 | 1.519326 | 8.912176 | 0.002004 |
| RP3-337O18.9 | -1.12758 | 9.020646 | 0.002035 |
| RP11-616M22.5 | -1.80742 | 8.732448 | 0.002035 |
| CTD-2636A23.2 | 1.292796 | 9.181368 | 0.002077 |
| RP11-477H21.2 | 1.112774 | 9.269231 | 0.002082 |
| AC004988.1 | -1.51146 | 8.957123 | 0.00214 |
| RP11-19E11.1 | 1.284301 | 9.221916 | 0.002194 |
| RP11-578B16.1 | 1.878743 | 8.798462 | 0.002309 |
| CTD-2223O18.1 | 1.526332 | 9.041115 | 0.002327 |
| RP5-1120P11.1 | 1.983326 | 9.345285 | 0.002366 |
| SENCR | -1.20888 | 8.988861 | 0.002518 |
| CTD-2171N6.1 | 2.129143 | 8.82839 | 0.002649 |
| AC034243.1 | 1.055821 | 9.251972 | 0.002856 |
| AC106869.2 | 1.658239 | 9.24231 | 0.002966 |
| LINC00482 | 1.322898 | 9.418693 | 0.003003 |
| LINC00461 | 1.394565 | 8.915146 | 0.003051 |
| RP11-116D17.3 | 1.293584 | 11.7201 | 0.00309 |
| CTA-243E7.2 | 2.387665 | 8.802156 | 0.003141 |
| CTD-2510F5.4 | 1.00207 | 9.472433 | 0.003354 |
| RP11-109M17.2 | 2.470916 | 9.628341 | 0.003447 |
| AP000473.5 | -1.18038 | 8.975508 | 0.003464 |
| RP11-54O7.1 | 1.399599 | 8.91385 | 0.003481 |
| RP11-588K22.2 | -1.07896 | 9.768832 | 0.003542 |
| RP1-293L6.1 | 1.894669 | 9.22377 | 0.003639 |
| LINC01451 | 1.88615 | 8.766193 | 0.003733 |
| RP11-857B24.5 | 1.203151 | 9.337562 | 0.003814 |
| AC141928.1 | 1.483739 | 9.153021 | 0.003939 |
| CTD-2003C8.2 | 2.02917 | 8.753544 | 0.004044 |
| HCG11 | -1.0392 | 9.070537 | 0.004062 |
| RP11-119J18.1 | -1.663 | 9.066895 | 0.004068 |
| LINC00278 | -1.39877 | 8.687022 | 0.004222 |
| RP11-35O7.1 | 2.233083 | 8.776832 | 0.004228 |
| AC011239.1 | 1.087345 | 9.524096 | 0.004235 |
| RP11-93K22.6 | 1.654117 | 8.755558 | 0.004246 |
| RP11-1041F24.1 | 1.840924 | 8.908628 | 0.004327 |
| RP11-642C5.1 | -1.27117 | 8.937558 | 0.004399 |
| LINC01436 | 2.397058 | 9.114892 | 0.004446 |
| RP11-260M19.2 | 1.130469 | 9.338587 | 0.004495 |
| RP11-167B3.2 | -1.83699 | 8.901975 | 0.004663 |
| LINC00604 | 1.980872 | 8.74217 | 0.004676 |
| CTA-228A9.3 | 1.090525 | 9.104507 | 0.004838 |
| RP11-430E17.1 | 1.469591 | 8.755744 | 0.004955 |
| AC093850.2 | 1.95382 | 8.793105 | 0.00512 |
| DIO3OS | -1.61757 | 8.780415 | 0.005144 |
| AF131216.5 | 1.167067 | 8.876458 | 0.005244 |
| RP11-750H9.5 | -1.22167 | 8.892638 | 0.005254 |
| RP11-295M3.4 | -1.21793 | 8.98432 | 0.005298 |
| RP11-395N3.2 | 1.345254 | 9.438646 | 0.005314 |
| LINC01489 | -1.50362 | 8.763332 | 0.005362 |
| LINC00519 | 1.351083 | 8.948754 | 0.005403 |
| CATIP-AS2 | 1.837468 | 8.936985 | 0.005441 |
| RP11-211G23.2 | 2.063382 | 8.827751 | 0.00547 |
| GS1-594A7.3 | 1.646399 | 9.101599 | 0.005536 |
| CTD-2008P7.9 | -1.68595 | 8.711953 | 0.005743 |
| RP11-61L19.2 | 1.462343 | 9.14478 | 0.005795 |
| KLF3-AS1 | 1.026814 | 9.100142 | 0.005911 |
| LINC00540 | 1.069577 | 9.227871 | 0.006012 |
| RP11-753H16.5 | -1.32737 | 8.798018 | 0.006201 |
| LINC01550 | 1.641203 | 8.848389 | 0.006217 |
| AP001437.1 | 1.064031 | 9.126063 | 0.006302 |
| AC007255.8 | 1.360527 | 8.755959 | 0.0064 |
| LINC00087 | -1.07959 | 12.66705 | 0.006537 |
| RP11-493L12.5 | 1.570207 | 8.76482 | 0.006857 |
| AC090044.2 | -1.71286 | 8.769587 | 0.007002 |
| AC023137.2 | 1.174938 | 8.920302 | 0.007046 |
| RP11-148L24.1 | 1.693216 | 8.762368 | 0.00722 |
| AC004381.7 | -1.39952 | 8.876621 | 0.007385 |
| RP11-545N8.3 | -1.61339 | 9.167948 | 0.007423 |
| LINC00619 | 2.154337 | 8.792401 | 0.007592 |
| TBX2-AS1 | 1.09439 | 9.474108 | 0.007688 |
| RP11-473L15.3 | 1.573728 | 8.788218 | 0.007739 |
| CTD-2525I3.2 | 1.021584 | 9.045287 | 0.007863 |
| RP11-261N11.8 | 1.643819 | 8.792721 | 0.00807 |
| AC004449.6 | 1.21283 | 8.841034 | 0.008228 |
| HOXB-AS4 | 1.323578 | 9.017516 | 0.008235 |
| RP5-1011O1.2 | 1.483447 | 9.139178 | 0.008302 |
| AC011516.2 | -1.6641 | 8.704105 | 0.008376 |
| LINC00377 | 1.480882 | 8.810163 | 0.008569 |
| FAM225A | 1.36013 | 8.749277 | 0.008632 |
| RP11-219E7.1 | -1.05934 | 8.978989 | 0.008722 |
| RP11-417L19.2 | 1.480345 | 8.841314 | 0.008764 |
| RP5-1024C24.1 | -1.18988 | 8.807274 | 0.008837 |
| LINC01537 | 1.186714 | 9.447354 | 0.008952 |
| RP11-1101K5.1 | 1.016916 | 9.182361 | 0.008954 |
| RP11-1114I9.1 | 1.290426 | 8.81874 | 0.008957 |
| CTD-2526A2.2 | 1.11726 | 9.433786 | 0.008985 |
| RP11-218F4.1 | 1.544582 | 8.760165 | 0.009141 |
| RP11-410N8.3 | -1.31766 | 9.067124 | 0.009176 |
| AC016629.3 | 1.650118 | 8.74602 | 0.009392 |
| LINC00523 | 1.555111 | 8.973145 | 0.010202 |
| SERHL | 1.107319 | 8.944182 | 0.010624 |
| LINC01540 | 1.48766 | 8.910468 | 0.01066 |
| RP11-495O11.1 | 1.583497 | 8.778243 | 0.010758 |
| AC016745.3 | -1.23903 | 8.758057 | 0.0109 |
| RP11-25K19.1 | 1.33665 | 8.861896 | 0.010983 |
| CTD-2396E7.11 | -1.26026 | 9.055938 | 0.011274 |
| OVAAL | -1.58417 | 8.695477 | 0.011441 |
| TSSC1-IT1 | 1.003665 | 9.091651 | 0.011477 |
| TTTY15 | -1.30931 | 8.863442 | 0.011567 |
| RP11-536G4.1 | 1.136132 | 8.912261 | 0.011605 |
| RP11-203E8.1 | -1.57572 | 8.687766 | 0.011627 |
| RP11-197K6.1 | 1.874872 | 9.65912 | 0.011742 |
| RP11-715J22.6 | 1.469605 | 8.790433 | 0.01228 |
| CTD-3012A18.1 | 1.45753 | 8.810336 | 0.012642 |
| FLJ42969 | -1.29513 | 8.780767 | 0.013177 |
| KB-1980E6.3 | 1.364171 | 8.718516 | 0.013297 |
| RP11-552D4.1 | -1.1206 | 9.0364 | 0.013383 |
| RP11-734I18.1 | -1.54744 | 8.767788 | 0.01353 |
| AC013275.2 | -1.55192 | 8.759655 | 0.014067 |
| RP11-123K19.1 | 1.216903 | 8.807131 | 0.014072 |
| POT1-AS1 | -1.02727 | 8.907153 | 0.014216 |
| PDX1-AS1 | 1.449664 | 8.691625 | 0.014443 |
| RP11-179K3.2 | -1.4076 | 8.677363 | 0.014875 |
| CTD-2357A8.3 | 1.888023 | 8.743723 | 0.014902 |
| CTD-2561B21.5 | 1.194651 | 9.421982 | 0.015136 |
| RP11-594C13.1 | 1.371244 | 8.782922 | 0.015287 |
| TRIM31-AS1 | -1.02265 | 8.919818 | 0.015333 |
| AC067959.1 | -1.64581 | 8.827668 | 0.01568 |
| LA16c-329F2.1 | 1.670552 | 8.809119 | 0.01568 |
| RP11-677M24.1 | 1.260031 | 8.871483 | 0.015998 |
| RP5-875H18.10 | -1.1626 | 8.771636 | 0.016423 |
| CTD-2341M24.1 | 1.041414 | 9.279716 | 0.016475 |
| FEZF1-AS1 | 1.409444 | 8.821189 | 0.016481 |
| FOXD3-AS1 | 1.164423 | 9.286622 | 0.01666 |
| RP11-424G14.1 | -1.19196 | 8.771317 | 0.016721 |
| ZFHX4-AS1 | 1.072291 | 9.094979 | 0.016752 |
| RP11-114H21.2 | 1.436147 | 8.730015 | 0.016917 |
| BX322559.3 | 2.264626 | 9.226289 | 0.017064 |
| HOXA10-AS | 1.350669 | 8.71168 | 0.017242 |
| SSTR5-AS1 | -1.54688 | 11.05223 | 0.018407 |
| CCDC144NL-AS1 | 1.465451 | 8.738928 | 0.018826 |
| LA16c-352F7.1 | -1.21762 | 8.83144 | 0.02046 |
| HOTAIR | 1.336163 | 8.769128 | 0.020698 |
| RP11-74E22.8 | 1.055942 | 9.004683 | 0.021099 |
| AC068580.7 | 1.462809 | 8.690756 | 0.021103 |
| RP11-476M19.2 | -1.66118 | 8.728174 | 0.021784 |
| RP4-547N15.3 | -1.45844 | 8.942742 | 0.022868 |
| RP11-429J17.7 | 1.087374 | 8.794374 | 0.022953 |
| RP11-65L3.4 | 1.052116 | 8.989654 | 0.023234 |
| RP11-44N21.1 | 1.095555 | 8.908066 | 0.023597 |
| RP11-673F18.1 | 1.222837 | 8.797654 | 0.023883 |
| RP11-11N9.4 | -1.02154 | 9.514986 | 0.023905 |
| RP1-170O19.17 | 1.015661 | 9.042756 | 0.023977 |
| RP11-461A8.4 | 1.222586 | 8.855839 | 0.024115 |
| LINC00840 | 1.470785 | 8.733115 | 0.024501 |
| RP11-703H8.9 | 1.529783 | 8.772859 | 0.024733 |
| CTD-2525I3.3 | 1.205414 | 8.736745 | 0.025539 |
| RP11-757G1.6 | 1.204475 | 8.815179 | 0.025905 |
| RP11-100E13.1 | 1.694226 | 8.843714 | 0.025938 |
| RP11-116D17.5 | 1.173774 | 9.858997 | 0.026759 |
| RP11-431J24.2 | -1.0187 | 9.011884 | 0.027073 |
| AC006552.1 | 1.373161 | 9.115671 | 0.027261 |
| LINC00332 | 1.277364 | 8.88788 | 0.02739 |
| RP11-468N14.13 | -1.54206 | 8.686182 | 0.027454 |
| IDI2-AS1 | 1.009855 | 8.885427 | 0.027501 |
| RP11-700N1.1 | 1.44211 | 8.751593 | 0.027547 |
| RP11-148M9.1 | 1.792285 | 8.706601 | 0.027615 |
| RP11-575F12.2 | 1.10954 | 8.976637 | 0.027983 |
| CTD-2555A7.2 | 1.408969 | 8.68458 | 0.028562 |
| RP5-884M6.1 | 1.223929 | 8.827169 | 0.029587 |
| RP11-95I16.6 | -1.38179 | 8.676084 | 0.030399 |
| RP11-150O12.3 | -1.42962 | 8.879333 | 0.030625 |
| AC114730.5 | -1.4187 | 8.794291 | 0.031835 |
| CTC-501O10.1 | -1.09453 | 9.146866 | 0.032403 |
| TET2-AS1 | 1.183303 | 8.787294 | 0.032876 |
| RP3-329E20.2 | 1.042548 | 8.862366 | 0.033233 |
| RP11-797A18.6 | 1.064705 | 8.821436 | 0.034612 |
| LINC01272 | 1.124994 | 8.932834 | 0.03468 |
| RP11-775B15.2 | 1.263554 | 8.766192 | 0.03527 |
| RP11-467I17.1 | -1.13135 | 8.68652 | 0.035405 |
| RP3-413H6.2 | 1.178476 | 8.779932 | 0.035728 |
| LRP4-AS1 | 1.240176 | 8.889217 | 0.035942 |
| AC010084.1 | -1.30103 | 8.715158 | 0.036492 |
| AC073409.1 | -1.6432 | 8.785881 | 0.03665 |
| LINC01150 | -1.00278 | 8.829056 | 0.036806 |
| AC010907.5 | 1.178487 | 8.708157 | 0.037457 |
| MIR31HG | 1.307987 | 8.699938 | 0.037928 |
| RP11-525A16.4 | 1.030754 | 8.835738 | 0.037932 |
| RP11-83J21.3 | 1.277826 | 8.789251 | 0.039031 |
| RP11-103J17.2 | 1.020395 | 8.832411 | 0.039417 |
| RP11-314N14.1 | 1.19607 | 8.672974 | 0.039427 |
| AC112715.2 | 1.205756 | 8.794113 | 0.039543 |
| LA16c-380H5.4 | 1.01909 | 8.784342 | 0.040253 |
| AC004471.9 | 1.089159 | 8.83844 | 0.040808 |
| RP11-74M11.2 | -1.28911 | 8.699606 | 0.041246 |
| AC027119.1 | -1.26818 | 8.703172 | 0.04234 |
| AFAP1-AS1 | -1.39294 | 8.716368 | 0.042565 |
| RP4-597A16.2 | 1.13533 | 8.717782 | 0.043123 |
| RP11-1406H17.1 | 1.098251 | 8.695961 | 0.043123 |
| CTD-2081C10.1 | 1.02118 | 8.706924 | 0.043123 |
| RP11-369E15.3 | 1.128854 | 9.252029 | 0.043174 |
| RP11-495P10.8 | 1.136207 | 8.73501 | 0.04326 |
| RP13-895J2.7 | 1.208216 | 8.718347 | 0.043267 |
| CTC-241F20.4 | 1.149113 | 8.729491 | 0.043267 |
| RP11-523O18.5 | 1.098032 | 8.729948 | 0.043267 |
| RP11-816J6.3 | 1.084005 | 8.732681 | 0.043267 |
| CTD-2647L4.1 | 1.241929 | 8.675988 | 0.044509 |
| RP11-885N19.6 | 1.145627 | 8.66466 | 0.044888 |
| RP1-240B8.3 | -1.26256 | 8.806661 | 0.045096 |
| MYHAS | 1.196644 | 8.669478 | 0.046265 |
| RP11-247C2.2 | 1.134849 | 8.691415 | 0.046573 |
| CTD-2314G24.2 | 1.024159 | 8.704091 | 0.046786 |
| AATBC | 1.145719 | 8.76932 | 0.047424 |
| RP13-650J16.1 | 1.025354 | 8.80183 | 0.047964 |

**Supplementary Table S4**

**All differential expression miRNAs in the cluster2 compared to cluster1**

| miRNA | logFC | logCPM | PValue |
| --- | --- | --- | --- |
| hsa-mir-183 | 3.270979 | 13.65957 | 9.29E-10 |
| hsa-mir-653 | -2.24085 | 4.150177 | 1.47E-09 |
| hsa-mir-182 | 2.860673 | 14.94417 | 4.28E-09 |
| hsa-mir-330 | 1.4645 | 5.496134 | 3.96E-08 |
| hsa-mir-96 | 2.623757 | 5.127494 | 4.26E-08 |
| hsa-mir-1247 | -3.32107 | 4.678056 | 5.04E-07 |
| hsa-mir-194-1 | -2.15275 | 7.470433 | 5.78E-07 |
| hsa-mir-1258 | -2.49204 | 1.710481 | 7.64E-07 |
| hsa-mir-194-2 | -2.11413 | 7.720842 | 8.35E-07 |
| hsa-mir-31 | 5.104516 | 3.100668 | 1.12E-06 |
| hsa-mir-2115 | 4.085545 | -0.10509 | 1.77E-06 |
| hsa-mir-4800 | -2.48301 | -0.89154 | 2.72E-06 |
| hsa-mir-196b | 1.71229 | 7.601098 | 3.07E-06 |
| hsa-mir-5698 | 2.121347 | 0.11919 | 3.28E-06 |
| hsa-mir-489 | -2.93821 | 0.133069 | 3.46E-06 |
| hsa-mir-466 | -2.53759 | 2.12034 | 4.41E-06 |
| hsa-mir-146a | -1.49968 | 4.227575 | 8.07E-06 |
| hsa-mir-362 | -1.18926 | 5.694101 | 1.83E-05 |
| hsa-mir-1250 | 2.199298 | -0.79838 | 2.33E-05 |
| hsa-mir-513a-2 | -2.04927 | 5.521761 | 3.37E-05 |
| hsa-mir-1248 | 1.692922 | 2.541045 | 3.77E-05 |
| hsa-mir-6505 | -1.72121 | -0.53831 | 5.82E-05 |
| hsa-mir-513a-1 | -2.0494 | 5.431614 | 5.88E-05 |
| hsa-mir-6788 | -1.98879 | -0.37551 | 6.18E-05 |
| hsa-mir-196a-1 | 1.832812 | 5.08374 | 6.45E-05 |
| hsa-mir-4449 | 1.918605 | 1.236376 | 9.37E-05 |
| hsa-mir-223 | 1.667595 | 7.653902 | 0.000106 |
| hsa-mir-3170 | 1.314814 | 1.430153 | 0.000119 |
| hsa-mir-548az | 4.627646 | -0.44947 | 0.000133 |
| hsa-mir-4423 | -1.86551 | 2.997731 | 0.000146 |
| hsa-mir-196a-2 | 1.676009 | 5.769757 | 0.000183 |
| hsa-mir-3065 | 1.450862 | 5.962756 | 0.000213 |
| hsa-mir-4501 | -1.80535 | 1.205884 | 0.000262 |
| hsa-mir-7114 | 3.423818 | -0.50977 | 0.000286 |
| hsa-mir-150 | -1.35589 | 6.947355 | 0.000327 |
| hsa-mir-99a | -1.147 | 9.212535 | 0.000413 |
| hsa-mir-218-2 | 1.379861 | 6.315643 | 0.000479 |
| hsa-mir-510 | -1.66413 | 3.902122 | 0.000522 |
| hsa-mir-3940 | 1.315517 | 0.663957 | 0.000645 |
| hsa-mir-218-1 | 1.350894 | 6.378938 | 0.000713 |
| hsa-mir-6720 | 2.749606 | 0.299181 | 0.000862 |
| hsa-mir-378d-2 | -1.24463 | -0.02786 | 0.000914 |
| hsa-mir-95 | 1.229833 | 3.898811 | 0.001007 |
| hsa-mir-92b | 1.116484 | 6.021111 | 0.001056 |
| hsa-mir-514a-2 | -1.57479 | 11.82036 | 0.001121 |
| hsa-mir-514a-3 | -1.57298 | 11.81752 | 0.001126 |
| hsa-mir-514a-1 | -1.57325 | 11.81819 | 0.001129 |
| hsa-mir-3132 | 1.76998 | -0.93566 | 0.001304 |
| hsa-mir-211 | -2.442 | -0.85301 | 0.001307 |
| hsa-mir-214 | 1.274576 | 3.123033 | 0.001403 |
| hsa-mir-508 | -1.48683 | 14.75858 | 0.0016 |
| hsa-mir-3190 | 1.217093 | -0.21919 | 0.001941 |
| hsa-mir-675 | 1.933947 | 7.604707 | 0.002002 |
| hsa-mir-4465 | 2.927347 | -0.5181 | 0.002051 |
| hsa-mir-6850 | 1.9322 | -1.01766 | 0.002177 |
| hsa-mir-6718 | -2.07284 | -0.22444 | 0.002291 |
| hsa-mir-873 | -2.45055 | 0.171615 | 0.002327 |
| hsa-mir-6757 | -1.30312 | -0.52174 | 0.002442 |
| hsa-mir-124-1 | 1.872716 | -0.45406 | 0.002518 |
| hsa-mir-4652 | 2.618576 | -0.99478 | 0.002555 |
| hsa-mir-3619 | 1.115089 | -0.15348 | 0.002703 |
| hsa-mir-192 | -1.20537 | 9.504009 | 0.002898 |
| hsa-mir-210 | 1.064676 | 10.29867 | 0.002971 |
| hsa-mir-1269b | 3.12251 | 4.338502 | 0.003081 |
| hsa-mir-139 | 1.420228 | 10.91327 | 0.003205 |
| hsa-mir-548ag-2 | -1.66988 | -0.28109 | 0.003222 |
| hsa-mir-219a-2 | -2.3186 | -0.40493 | 0.003362 |
| hsa-mir-6805 | 1.218311 | -0.53467 | 0.003391 |
| hsa-mir-3681 | 2.404777 | -0.80858 | 0.003626 |
| hsa-mir-605 | -1.23298 | 0.352839 | 0.00364 |
| hsa-mir-378d-1 | -1.13006 | -0.27281 | 0.004049 |
| hsa-mir-507 | -1.38131 | 5.913635 | 0.004113 |
| hsa-mir-7641-1 | 2.543404 | -0.99193 | 0.004222 |
| hsa-mir-506 | -1.38851 | 8.747645 | 0.004481 |
| hsa-mir-6715a | 1.435977 | -1.01135 | 0.004523 |
| hsa-mir-124-2 | 1.916766 | -0.36103 | 0.005169 |
| hsa-mir-9-3 | 1.410435 | 12.50025 | 0.00537 |
| hsa-mir-9-1 | 1.409606 | 12.49645 | 0.005414 |
| hsa-mir-9-2 | 1.407949 | 12.49713 | 0.005532 |
| hsa-mir-1193 | 1.247922 | -0.32373 | 0.005802 |
| hsa-mir-876 | -2.27559 | -0.34965 | 0.005973 |
| hsa-mir-7641-2 | 1.734596 | -0.77229 | 0.00608 |
| hsa-mir-513b | -1.31951 | 5.468133 | 0.006562 |
| hsa-mir-4433b | 1.459927 | -0.91122 | 0.007203 |
| hsa-mir-770 | 1.430301 | 1.266589 | 0.007365 |
| hsa-mir-6852 | 1.046419 | -0.58121 | 0.008129 |
| hsa-mir-3911 | 1.393604 | -0.92528 | 0.00863 |
| hsa-mir-187 | 1.425243 | 1.223264 | 0.008839 |
| hsa-mir-509-3 | -1.20726 | 11.20251 | 0.009598 |
| hsa-mir-4674 | 1.405098 | -0.9182 | 0.009724 |
| hsa-mir-3923 | 1.968136 | 0.663327 | 0.009774 |
| hsa-mir-124-3 | 1.835924 | -0.36712 | 0.011983 |
| hsa-mir-129-1 | 1.256579 | 3.524594 | 0.012395 |
| hsa-mir-4658 | 1.345138 | 0.186524 | 0.012522 |
| hsa-mir-373 | 2.271884 | -0.52168 | 0.012598 |
| hsa-mir-3176 | 1.126732 | -0.85215 | 0.012916 |
| hsa-mir-129-2 | 1.176886 | 3.489152 | 0.013665 |
| hsa-mir-3144 | 1.524652 | -0.21041 | 0.014089 |
| hsa-mir-217 | -1.35563 | 7.223261 | 0.01552 |
| hsa-mir-548f-1 | 1.488075 | -0.65782 | 0.015908 |
| hsa-mir-509-1 | -1.12402 | 10.9926 | 0.016099 |
| hsa-mir-3622a | 1.374363 | -1.11756 | 0.016224 |
| hsa-mir-509-2 | -1.12177 | 10.99223 | 0.016587 |
| hsa-mir-4424 | -1.39266 | -1.05777 | 0.017223 |
| hsa-mir-190b | -1.37717 | 0.155163 | 0.017572 |
| hsa-mir-4489 | 1.323781 | -1.16519 | 0.017722 |
| hsa-mir-6783 | 1.165236 | -0.45286 | 0.018094 |
| hsa-mir-6807 | 1.423398 | -0.85307 | 0.018467 |
| hsa-mir-3921 | -1.58004 | -1.15644 | 0.018835 |
| hsa-mir-6815 | 1.11234 | -0.42142 | 0.018999 |
| hsa-mir-134 | 1.120444 | 11.64224 | 0.019006 |
| hsa-mir-3924 | -1.38966 | -1.16668 | 0.019117 |
| hsa-mir-548x | 1.716856 | -1.13449 | 0.019227 |
| hsa-mir-4763 | 1.073336 | -0.11696 | 0.01939 |
| hsa-mir-1231 | 1.504743 | -1.09001 | 0.019787 |
| hsa-mir-4723 | -1.01273 | -0.88815 | 0.020403 |
| hsa-mir-1269a | 1.875018 | 8.440907 | 0.020985 |
| hsa-mir-1295b | -1.52032 | -0.77018 | 0.022799 |
| hsa-mir-6886 | 1.014154 | 0.201102 | 0.023072 |
| hsa-mir-1197 | 1.260492 | 1.977537 | 0.026547 |
| hsa-mir-370 | 1.099614 | 7.739619 | 0.026819 |
| hsa-mir-522 | -1.6379 | -1.04809 | 0.028983 |
| hsa-mir-3151 | -1.18198 | -0.85224 | 0.029112 |
| hsa-mir-4697 | -1.47389 | -1.1648 | 0.029163 |
| hsa-mir-592 | -1.17355 | 0.868245 | 0.029962 |
| hsa-mir-1246 | 1.518152 | -1.02323 | 0.030151 |
| hsa-mir-5694 | 1.554896 | -0.49915 | 0.031014 |
| hsa-mir-670 | 1.283717 | -0.54865 | 0.031327 |
| hsa-mir-1908 | 1.137329 | -0.90047 | 0.034392 |
| hsa-mir-1911 | 1.583503 | -0.93008 | 0.036003 |
| hsa-mir-3654 | 1.328964 | -0.59656 | 0.036454 |
| hsa-mir-3675 | 1.423529 | -1.10253 | 0.038337 |
| hsa-mir-4745 | 1.015899 | 0.50937 | 0.039059 |
| hsa-mir-383 | 1.691263 | 2.874777 | 0.04145 |
| hsa-mir-7703 | 1.263634 | -1.1336 | 0.043303 |
| hsa-mir-6860 | 1.585425 | -1.06033 | 0.04414 |
| hsa-mir-541 | 1.137002 | 4.956581 | 0.046613 |
| hsa-mir-5580 | 1.099514 | -0.95088 | 0.046755 |
| hsa-mir-4523 | 1.089164 | -0.98026 | 0.048233 |

**Supplementary Table S5**

**Targeted mRNAs base on miRTarBase, miRDB, and TargetScan databases.**

| miRNA | Gene | miRDB | miRTarBase | TargetScan | Sum |
| --- | --- | --- | --- | --- | --- |
| hsa-mir-373-3p | LEFTY1 | 1 | 1 | 1 | 3 |
| hsa-mir-223-3p | FOXO1 | 1 | 1 | 1 | 3 |
| hsa-mir-183-5p | PDCD4 | 1 | 1 | 1 | 3 |
| hsa-mir-223-3p | ECT2 | 1 | 1 | 1 | 3 |
| hsa-mir-31-5p | LATS2 | 1 | 1 | 1 | 3 |
| hsa-mir-217 | SIRT1 | 1 | 1 | 1 | 3 |
| hsa-mir-150-5p | CBL | 1 | 1 | 1 | 3 |
| hsa-mir-211-5p | SLC22A6 | 1 | 1 | 1 | 3 |
| hsa-mir-373-3p | SAR1B | 1 | 1 | 1 | 3 |
| hsa-mir-183-5p | EZR | 1 | 1 | 1 | 3 |
| hsa-mir-373-3p | WEE1 | 1 | 1 | 1 | 3 |
| hsa-mir-214-3p | LZTS1 | 1 | 1 | 1 | 3 |
| hsa-mir-214-3p | PAPPA | 1 | 1 | 1 | 3 |
| hsa-mir-506-3p | ZWINT | 1 | 1 | 1 | 3 |
| hsa-mir-223-3p | CFTR | 1 | 1 | 1 | 3 |
| hsa-mir-373-3p | TGFBR2 | 1 | 1 | 1 | 3 |
| hsa-mir-183-5p | TMED7 | 1 | 1 | 1 | 3 |
| hsa-mir-183-5p | FAM175B | 1 | 1 | 1 | 3 |
| hsa-mir-182-5p | ADCY6 | 1 | 1 | 1 | 3 |
| hsa-mir-373-3p | PFKP | 1 | 1 | 1 | 3 |
| hsa-mir-373-3p | ZFYVE26 | 1 | 1 | 1 | 3 |
| hsa-mir-182-5p | NPTX1 | 1 | 1 | 1 | 3 |
| hsa-mir-31-5p | NOL9 | 1 | 1 | 1 | 3 |
| hsa-mir-211-5p | SYNJ2BP | 1 | 1 | 1 | 3 |
| hsa-mir-217 | FOXO3 | 1 | 1 | 1 | 3 |
| hsa-mir-373-3p | FEM1C | 1 | 1 | 1 | 3 |
| hsa-mir-96-5p | SNX7 | 1 | 1 | 1 | 3 |
| hsa-mir-211-5p | CHRDL1 | 1 | 1 | 1 | 3 |
| hsa-mir-183-5p | GLUL | 1 | 1 | 1 | 3 |
| hsa-mir-31-5p | NUMB | 1 | 1 | 1 | 3 |
| hsa-mir-192-5p | RB1 | 1 | 1 | 1 | 3 |
| hsa-mir-223-3p | IGF1R | 1 | 1 | 1 | 3 |
| hsa-mir-211-5p | ARAP2 | 1 | 1 | 1 | 3 |
| hsa-mir-373-3p | MPP5 | 1 | 1 | 1 | 3 |
| hsa-mir-214-3p | DOCK9 | 1 | 1 | 1 | 3 |
| hsa-mir-373-3p | ARHGEF3 | 1 | 1 | 1 | 3 |
| hsa-mir-506-3p | SLC16A1 | 1 | 1 | 1 | 3 |
| hsa-mir-373-3p | RHOC | 1 | 1 | 1 | 3 |
| hsa-mir-211-5p | SPOP | 1 | 1 | 1 | 3 |
| hsa-mir-183-5p | CCNB1 | 1 | 1 | 1 | 3 |
| hsa-mir-223-3p | RHOB | 1 | 1 | 1 | 3 |
| hsa-mir-96-5p | JAZF1 | 1 | 1 | 1 | 3 |
| hsa-mir-210-3p | POU2AF1 | 1 | 1 | 1 | 3 |
| hsa-mir-223-3p | FOXO3 | 1 | 1 | 1 | 3 |
| hsa-mir-214-3p | TWF1 | 1 | 1 | 1 | 3 |
| hsa-mir-373-3p | SUCO | 1 | 1 | 1 | 3 |
| hsa-mir-214-3p | QKI | 1 | 1 | 1 | 3 |
| hsa-mir-223-3p | FBXW7 | 1 | 1 | 1 | 3 |
| hsa-mir-373-3p | INO80D | 1 | 1 | 1 | 3 |
| hsa-mir-182-5p | HOXA9 | 1 | 1 | 1 | 3 |
| hsa-mir-214-3p | LTF | 1 | 1 | 1 | 3 |
| hsa-mir-217 | TNFRSF21 | 1 | 1 | 1 | 3 |
| hsa-mir-211-5p | IL11 | 1 | 1 | 1 | 3 |
| hsa-mir-211-5p | PRDM2 | 1 | 1 | 1 | 3 |
| hsa-mir-96-5p | KRAS | 1 | 1 | 1 | 3 |
| hsa-mir-192-5p | RPAP2 | 1 | 1 | 1 | 3 |
| hsa-mir-192-5p | ZMAT3 | 1 | 1 | 1 | 3 |
| hsa-mir-373-3p | FBXL7 | 1 | 1 | 1 | 3 |
| hsa-mir-150-5p | AMOTL2 | 1 | 1 | 1 | 3 |
| hsa-mir-214-3p | FAM49B | 1 | 1 | 1 | 3 |
| hsa-mir-211-5p | ZBTB22 | 1 | 1 | 1 | 3 |
| hsa-mir-210-3p | DENND6A | 1 | 1 | 1 | 3 |
| hsa-mir-192-5p | ZDHHC2 | 1 | 1 | 1 | 3 |
| hsa-mir-211-5p | IKZF2 | 1 | 1 | 1 | 3 |
| hsa-mir-150-5p | PLEKHM3 | 1 | 1 | 1 | 3 |
| hsa-mir-214-3p | PSMD10 | 1 | 1 | 1 | 3 |
| hsa-mir-31-5p | SELE | 1 | 1 | 1 | 3 |
| hsa-mir-182-5p | MITF | 1 | 1 | 1 | 3 |
| hsa-mir-31-5p | ARID1A | 1 | 1 | 1 | 3 |
| hsa-mir-383-5p | IRF1 | 1 | 1 | 1 | 3 |
| hsa-mir-150-5p | XPNPEP3 | 1 | 1 | 1 | 3 |
| hsa-mir-210-3p | ALDH5A1 | 1 | 1 | 1 | 3 |
| hsa-mir-96-5p | DDIT3 | 1 | 1 | 1 | 3 |
| hsa-mir-192-5p | GRHL1 | 1 | 1 | 1 | 3 |
| hsa-mir-223-3p | CDC27 | 1 | 1 | 1 | 3 |
| hsa-mir-211-5p | ITPR1 | 1 | 1 | 1 | 3 |
| hsa-mir-373-3p | OSTM1 | 1 | 1 | 1 | 3 |
| hsa-mir-223-3p | RIF1 | 1 | 1 | 1 | 3 |
| hsa-mir-214-3p | CAPN5 | 1 | 1 | 1 | 3 |
| hsa-mir-214-3p | MAPK1 | 1 | 1 | 1 | 3 |
| hsa-mir-192-5p | NCOA3 | 1 | 1 | 1 | 3 |
| hsa-mir-183-5p | ITGB1 | 1 | 1 | 1 | 3 |
| hsa-mir-96-5p | ABCD1 | 1 | 1 | 1 | 3 |
| hsa-mir-192-5p | CTNNBIP1 | 1 | 1 | 1 | 3 |
| hsa-mir-182-5p | KDM5A | 1 | 1 | 1 | 3 |
| hsa-mir-183-5p | PDCD6 | 1 | 1 | 1 | 3 |
| hsa-mir-183-5p | PPP2CB | 1 | 1 | 1 | 3 |
| hsa-mir-150-5p | EGR2 | 1 | 1 | 1 | 3 |
| hsa-mir-223-3p | ZNF365 | 1 | 1 | 1 | 3 |
| hsa-mir-373-3p | HIP1 | 1 | 1 | 1 | 3 |
| hsa-mir-373-3p | IRF2 | 1 | 1 | 1 | 3 |
| hsa-mir-217 | SNRNP27 | 1 | 1 | 1 | 3 |
| hsa-mir-183-5p | LRP6 | 1 | 1 | 1 | 3 |
| hsa-mir-373-3p | DPP8 | 1 | 1 | 1 | 3 |
| hsa-mir-211-5p | TCF12 | 1 | 1 | 1 | 3 |
| hsa-mir-210-3p | ISCU | 1 | 1 | 1 | 3 |
| hsa-mir-182-5p | BRWD1 | 1 | 1 | 1 | 3 |
| hsa-mir-96-5p | MORF4L1 | 1 | 1 | 1 | 3 |
| hsa-mir-31-5p | PPP2R2A | 1 | 1 | 1 | 3 |
| hsa-mir-211-5p | ELOVL6 | 1 | 1 | 1 | 3 |
| hsa-mir-223-3p | PAX6 | 1 | 1 | 1 | 3 |
| hsa-mir-182-5p | MTSS1 | 1 | 1 | 1 | 3 |
| hsa-mir-183-5p | SUCO | 1 | 1 | 1 | 3 |
| hsa-mir-211-5p | PRLR | 1 | 1 | 1 | 3 |
| hsa-mir-506-3p | LRRC1 | 1 | 1 | 1 | 3 |
| hsa-mir-506-3p | NEK9 | 1 | 1 | 1 | 3 |
| hsa-mir-31-5p | KLF13 | 1 | 1 | 1 | 3 |
| hsa-mir-31-5p | CCNT1 | 1 | 1 | 1 | 3 |
| hsa-mir-150-5p | ZEB1 | 1 | 1 | 1 | 3 |
| hsa-mir-96-5p | REV1 | 1 | 1 | 1 | 3 |
| hsa-mir-211-5p | SGPL1 | 1 | 1 | 1 | 3 |
| hsa-mir-183-5p | KLHL24 | 1 | 1 | 1 | 3 |
| hsa-mir-214-3p | ERC1 | 1 | 1 | 1 | 3 |
| hsa-mir-31-5p | PARP1 | 1 | 1 | 1 | 3 |
| hsa-mir-31-5p | SYDE2 | 1 | 1 | 1 | 3 |
| hsa-mir-31-5p | JAZF1 | 1 | 1 | 1 | 3 |
| hsa-mir-211-5p | CHORDC1 | 1 | 1 | 1 | 3 |
| hsa-mir-150-5p | SP1 | 1 | 1 | 1 | 3 |
| hsa-mir-214-3p | PPARGC1B | 1 | 1 | 1 | 3 |
| hsa-mir-214-3p | ZBTB10 | 1 | 1 | 1 | 3 |
| hsa-mir-373-3p | GNB5 | 1 | 1 | 1 | 3 |
| hsa-mir-214-3p | MPDU1 | 1 | 1 | 1 | 3 |
| hsa-mir-96-5p | CASP2 | 1 | 1 | 1 | 3 |
| hsa-mir-211-5p | SH3PXD2A | 1 | 1 | 1 | 3 |
| hsa-mir-192-5p | LIMS1 | 1 | 1 | 1 | 3 |
| hsa-mir-150-5p | MUC4 | 1 | 1 | 1 | 3 |
| hsa-mir-31-5p | ZC3H12C | 1 | 1 | 1 | 3 |
| hsa-mir-506-3p | SNAI2 | 1 | 1 | 1 | 3 |
| hsa-mir-214-3p | ZNF641 | 1 | 1 | 1 | 3 |
| hsa-mir-150-5p | PLXDC1 | 1 | 1 | 1 | 3 |
| hsa-mir-183-5p | GNG5 | 1 | 1 | 1 | 3 |
| hsa-mir-210-3p | VAMP4 | 1 | 1 | 1 | 3 |
| hsa-mir-31-5p | RHOBTB1 | 1 | 1 | 1 | 3 |
| hsa-mir-211-5p | TMTC2 | 1 | 1 | 1 | 3 |
| hsa-mir-31-5p | RASA1 | 1 | 1 | 1 | 3 |
| hsa-mir-373-3p | IGF1R | 1 | 1 | 1 | 3 |
| hsa-mir-183-5p | RCN2 | 1 | 1 | 1 | 3 |
| hsa-mir-210-3p | GPD1L | 1 | 1 | 1 | 3 |
| hsa-mir-211-5p | IGF2R | 1 | 1 | 1 | 3 |
| hsa-mir-223-3p | PHF19 | 1 | 1 | 1 | 3 |
| hsa-mir-183-5p | KLRD1 | 1 | 1 | 1 | 3 |
| hsa-mir-373-3p | LATS2 | 1 | 1 | 1 | 3 |
| hsa-mir-183-5p | CTDSPL | 1 | 1 | 1 | 3 |
| hsa-mir-373-3p | HMBOX1 | 1 | 1 | 1 | 3 |
| hsa-mir-373-3p | BTG1 | 1 | 1 | 1 | 3 |
| hsa-mir-150-5p | QSOX1 | 1 | 1 | 1 | 3 |
| hsa-mir-192-5p | TMED10 | 1 | 1 | 1 | 3 |
| hsa-mir-192-5p | PKP4 | 1 | 1 | 1 | 3 |
| hsa-mir-192-5p | ARL2BP | 1 | 1 | 1 | 3 |
| hsa-mir-96-5p | SNX16 | 1 | 1 | 1 | 3 |
| hsa-mir-383-5p | DIO1 | 1 | 1 | 1 | 3 |
| hsa-mir-214-3p | ARL2 | 1 | 1 | 1 | 3 |
| hsa-mir-214-3p | C17orf49 | 1 | 1 | 1 | 3 |
| hsa-mir-182-5p | FLOT1 | 1 | 1 | 1 | 3 |
| hsa-mir-214-3p | C10orf76 | 1 | 1 | 1 | 3 |
| hsa-mir-96-5p | TSKU | 1 | 1 | 1 | 3 |
| hsa-mir-96-5p | MED1 | 1 | 1 | 1 | 3 |
| hsa-mir-31-5p | NF2 | 1 | 1 | 1 | 3 |
| hsa-mir-211-5p | NPTX1 | 1 | 1 | 1 | 3 |
| hsa-mir-96-5p | ZEB1 | 1 | 1 | 1 | 3 |
| hsa-mir-506-3p | CHSY1 | 1 | 1 | 1 | 3 |
| hsa-mir-192-5p | ANAPC10 | 1 | 1 | 1 | 3 |
| hsa-mir-192-5p | ALCAM | 1 | 1 | 1 | 3 |
| hsa-mir-223-3p | IL6ST | 1 | 1 | 1 | 3 |
| hsa-mir-211-5p | AP1S2 | 1 | 1 | 1 | 3 |
| hsa-mir-182-5p | SESN2 | 1 | 1 | 1 | 3 |
| hsa-mir-96-5p | MBD4 | 1 | 1 | 1 | 3 |
| hsa-mir-183-5p | AKAP12 | 1 | 1 | 1 | 3 |
| hsa-mir-150-5p | TRPS1 | 1 | 1 | 1 | 3 |
| hsa-mir-211-5p | MBNL1 | 1 | 1 | 1 | 3 |
| hsa-mir-214-3p | VAV2 | 1 | 1 | 1 | 3 |
| hsa-mir-506-3p | GXYLT1 | 1 | 1 | 1 | 3 |
| hsa-mir-373-3p | PDIK1L | 1 | 1 | 1 | 3 |
| hsa-mir-373-3p | TXNIP | 1 | 1 | 1 | 3 |
| hsa-mir-210-3p | AIFM3 | 1 | 1 | 1 | 3 |
| hsa-mir-373-3p | TBC1D2 | 1 | 1 | 1 | 3 |
| hsa-mir-214-3p | PTEN | 1 | 1 | 1 | 3 |
| hsa-mir-31-5p | FOXD4L4 | 1 | 1 | 1 | 3 |
| hsa-mir-182-5p | FBXW7 | 1 | 1 | 1 | 3 |
| hsa-mir-182-5p | FOXO3 | 1 | 1 | 1 | 3 |
| hsa-mir-182-5p | CITED2 | 1 | 1 | 1 | 3 |
| hsa-mir-217 | PPM1D | 1 | 1 | 1 | 3 |
| hsa-mir-192-5p | FHDC1 | 1 | 1 | 1 | 3 |
| hsa-mir-182-5p | RARG | 1 | 1 | 1 | 3 |
| hsa-mir-373-3p | UNK | 1 | 1 | 1 | 3 |
| hsa-mir-182-5p | BDNF | 1 | 1 | 1 | 3 |
| hsa-mir-96-5p | FRS2 | 1 | 1 | 1 | 3 |
| hsa-mir-182-5p | CHL1 | 1 | 1 | 1 | 3 |
| hsa-mir-192-5p | DICER1 | 1 | 1 | 1 | 3 |
| hsa-mir-150-5p | MTMR9 | 1 | 1 | 1 | 3 |
| hsa-mir-31-5p | SP1 | 1 | 1 | 1 | 3 |
| hsa-mir-31-5p | YWHAE | 1 | 1 | 1 | 3 |
| hsa-mir-373-3p | ZNF385A | 1 | 1 | 1 | 3 |
| hsa-mir-373-3p | SUZ12 | 1 | 1 | 1 | 3 |
| hsa-mir-373-3p | NR2C2 | 1 | 1 | 1 | 3 |
| hsa-mir-183-5p | KIF5C | 1 | 1 | 1 | 3 |
| hsa-mir-223-3p | PTBP2 | 1 | 1 | 1 | 3 |
| hsa-mir-211-5p | ANGPTL2 | 1 | 1 | 1 | 3 |
| hsa-mir-506-3p | PRR14L | 1 | 1 | 1 | 3 |
| hsa-mir-506-3p | AMOTL1 | 1 | 1 | 1 | 3 |
| hsa-mir-192-5p | WNK1 | 1 | 1 | 1 | 3 |
| hsa-mir-192-5p | PABPC4 | 1 | 1 | 1 | 3 |
| hsa-mir-150-5p | SLC7A11 | 1 | 1 | 1 | 3 |
| hsa-mir-150-5p | HILPDA | 1 | 1 | 1 | 3 |
| hsa-mir-210-3p | KCMF1 | 1 | 1 | 1 | 3 |
| hsa-mir-96-5p | CCNG1 | 1 | 1 | 1 | 3 |
| hsa-mir-182-5p | CYLD | 1 | 1 | 1 | 3 |
| hsa-mir-96-5p | EDEM1 | 1 | 1 | 1 | 3 |
| hsa-mir-31-5p | C19orf12 | 1 | 1 | 1 | 3 |
| hsa-mir-373-3p | MBD2 | 1 | 1 | 1 | 3 |
| hsa-mir-506-3p | PI4K2B | 1 | 1 | 1 | 3 |
| hsa-mir-373-3p | SBNO1 | 1 | 1 | 1 | 3 |
| hsa-mir-192-5p | CCDC121 | 1 | 1 | 1 | 3 |
| hsa-mir-373-3p | SLC22A23 | 1 | 1 | 1 | 3 |
| hsa-mir-214-3p | KCTD15 | 1 | 1 | 1 | 3 |
| hsa-mir-506-3p | MYO10 | 1 | 1 | 1 | 3 |
| hsa-mir-192-5p | RABGAP1 | 1 | 1 | 1 | 3 |
| hsa-mir-150-5p | KLHL21 | 1 | 1 | 1 | 3 |
| hsa-mir-223-3p | TWF1 | 1 | 1 | 1 | 3 |
| hsa-mir-211-5p | CREB5 | 1 | 1 | 1 | 3 |
| hsa-mir-373-3p | MINK1 | 1 | 1 | 1 | 3 |
| hsa-mir-223-3p | EPB41L3 | 1 | 1 | 1 | 3 |
| hsa-mir-373-3p | FOXJ2 | 1 | 1 | 1 | 3 |
| hsa-mir-373-3p | SERF1A | 1 | 1 | 1 | 3 |
| hsa-mir-150-5p | GAN | 1 | 1 | 1 | 3 |
| hsa-mir-150-5p | GOSR1 | 1 | 1 | 1 | 3 |
| hsa-mir-214-3p | LUZP1 | 1 | 1 | 1 | 3 |
| hsa-mir-214-3p | TRIM29 | 1 | 1 | 1 | 3 |
| hsa-mir-217 | MAP1B | 1 | 1 | 1 | 3 |
| hsa-mir-373-3p | ELAVL2 | 1 | 1 | 1 | 3 |
| hsa-mir-373-3p | SIK1 | 1 | 1 | 1 | 3 |
| hsa-mir-211-5p | TGFBR2 | 1 | 1 | 1 | 3 |
| hsa-mir-192-5p | ARFGEF1 | 1 | 1 | 1 | 3 |
| hsa-mir-150-5p | ELK1 | 1 | 1 | 1 | 3 |
| hsa-mir-183-5p | FOXO1 | 1 | 1 | 1 | 3 |
| hsa-mir-192-5p | NOD2 | 1 | 1 | 1 | 3 |
| hsa-mir-96-5p | ASH1L | 1 | 1 | 1 | 3 |
| hsa-mir-214-3p | TMEM248 | 1 | 1 | 1 | 3 |
| hsa-mir-211-5p | ZFHX3 | 1 | 1 | 1 | 3 |
| hsa-mir-150-5p | PDIA6 | 1 | 1 | 1 | 3 |
| hsa-mir-373-3p | RAB11FIP1 | 1 | 1 | 1 | 3 |
| hsa-mir-150-5p | MYB | 1 | 1 | 1 | 3 |
| hsa-mir-214-3p | JAG2 | 1 | 1 | 1 | 3 |
| hsa-mir-373-3p | CADM2 | 1 | 1 | 1 | 3 |
| hsa-mir-210-3p | ACVR1B | 1 | 1 | 1 | 3 |
| hsa-mir-214-3p | XBP1 | 1 | 1 | 1 | 3 |
| hsa-mir-373-3p | DAZAP2 | 1 | 1 | 1 | 3 |
| hsa-mir-214-3p | HDGF | 1 | 1 | 1 | 3 |
| hsa-mir-373-3p | PAK2 | 1 | 1 | 1 | 3 |
| hsa-mir-150-5p | GK5 | 1 | 1 | 1 | 3 |
| hsa-mir-211-5p | SSRP1 | 1 | 1 | 1 | 3 |
| hsa-mir-211-5p | SAMD5 | 1 | 1 | 1 | 3 |
| hsa-mir-214-3p | TRAF1 | 1 | 1 | 1 | 3 |
| hsa-mir-31-5p | HOXC13 | 1 | 1 | 1 | 3 |
| hsa-mir-96-5p | TMEM170B | 1 | 1 | 1 | 3 |
| hsa-mir-31-5p | ECHDC1 | 1 | 1 | 1 | 3 |
| hsa-mir-373-3p | GALNT3 | 1 | 1 | 1 | 3 |
| hsa-mir-31-5p | STK40 | 1 | 1 | 1 | 3 |
| hsa-mir-373-3p | FYCO1 | 1 | 1 | 1 | 3 |
| hsa-mir-506-3p | LRRC58 | 1 | 1 | 1 | 3 |
| hsa-mir-182-5p | PRKAA2 | 1 | 1 | 1 | 3 |
| hsa-mir-373-3p | SH3GLB1 | 1 | 1 | 1 | 3 |
| hsa-mir-183-5p | RALGDS | 1 | 1 | 1 | 3 |
| hsa-mir-373-3p | TNKS2 | 1 | 1 | 1 | 3 |
| hsa-mir-96-5p | FOXO1 | 1 | 1 | 1 | 3 |
| hsa-mir-192-5p | KIF5B | 1 | 1 | 1 | 3 |
| hsa-mir-150-5p | RNF165 | 1 | 1 | 1 | 3 |
| hsa-mir-506-3p | NUFIP2 | 1 | 1 | 1 | 3 |
| hsa-mir-31-5p | FOXD4L5 | 1 | 1 | 1 | 3 |
| hsa-mir-183-5p | FAM217B | 1 | 1 | 1 | 3 |
| hsa-mir-223-3p | HSP90B1 | 1 | 1 | 1 | 3 |
| hsa-mir-217 | TCF7L2 | 1 | 1 | 1 | 3 |
| hsa-mir-214-3p | CRKL | 1 | 1 | 1 | 3 |
| hsa-mir-214-3p | PNPLA6 | 1 | 1 | 1 | 3 |
| hsa-mir-214-3p | AHNAK2 | 1 | 1 | 1 | 3 |
| hsa-mir-373-3p | TMEM100 | 1 | 1 | 1 | 3 |
| hsa-mir-373-3p | NFIB | 1 | 1 | 1 | 3 |
| hsa-mir-506-3p | CREBRF | 1 | 1 | 1 | 3 |
| hsa-mir-214-3p | ALPK2 | 1 | 1 | 1 | 3 |
| hsa-mir-223-3p | FAM60A | 1 | 1 | 1 | 3 |
| hsa-mir-211-5p | ZCCHC24 | 1 | 1 | 1 | 3 |
| hsa-mir-211-5p | SP1 | 1 | 1 | 1 | 3 |
| hsa-mir-506-3p | VIM | 1 | 1 | 1 | 3 |
| hsa-mir-211-5p | PPP3R1 | 1 | 1 | 1 | 3 |
| hsa-mir-96-5p | SLC39A1 | 1 | 1 | 1 | 3 |
| hsa-mir-211-5p | PTPRT | 1 | 1 | 1 | 3 |
| hsa-mir-214-3p | NAP1L4 | 1 | 1 | 1 | 3 |
| hsa-mir-96-5p | CNNM3 | 1 | 1 | 1 | 3 |
| hsa-mir-210-3p | SH3BGRL | 1 | 1 | 1 | 3 |
| hsa-mir-373-3p | SERF1B | 1 | 1 | 1 | 3 |
| hsa-mir-150-5p | ADIPOR2 | 1 | 1 | 1 | 3 |
| hsa-mir-506-3p | SCAMP4 | 1 | 1 | 1 | 3 |
| hsa-mir-96-5p | ALK | 1 | 1 | 1 | 3 |
| hsa-mir-192-5p | ITGAV | 1 | 1 | 1 | 3 |
| hsa-mir-150-5p | EREG | 1 | 1 | 1 | 3 |
| hsa-mir-96-5p | PRKAR1A | 1 | 1 | 1 | 3 |
| hsa-mir-373-3p | ADAM9 | 1 | 1 | 1 | 3 |
| hsa-mir-373-3p | REST | 1 | 1 | 1 | 3 |
| hsa-mir-223-3p | RRAS2 | 1 | 1 | 1 | 3 |
| hsa-mir-31-5p | FZD3 | 1 | 1 | 1 | 3 |
| hsa-mir-31-5p | FOXD4 | 1 | 1 | 1 | 3 |
| hsa-mir-211-5p | CAPRIN1 | 1 | 1 | 1 | 3 |
| hsa-mir-223-3p | 2-Sep | 1 | 1 | 1 | 3 |
| hsa-mir-373-3p | CDK19 | 1 | 1 | 1 | 3 |
| hsa-mir-182-5p | CLOCK | 1 | 1 | 1 | 3 |
| hsa-mir-223-3p | PRDM1 | 1 | 1 | 1 | 3 |
| hsa-mir-211-5p | POU3F2 | 1 | 1 | 1 | 3 |
| hsa-mir-210-3p | SIN3A | 1 | 1 | 1 | 3 |
| hsa-mir-214-3p | ING4 | 1 | 1 | 1 | 3 |
| hsa-mir-192-5p | USP45 | 1 | 1 | 1 | 3 |
| hsa-mir-373-3p | SLAIN2 | 1 | 1 | 1 | 3 |
| hsa-mir-214-3p | GALNT7 | 1 | 1 | 1 | 3 |
| hsa-mir-217 | ADSS | 1 | 1 | 1 | 3 |
| hsa-mir-211-5p | WWC3 | 1 | 1 | 1 | 3 |
| hsa-mir-192-5p | SEMA4D | 1 | 1 | 1 | 3 |
| hsa-mir-211-5p | HNRNPA2B1 | 1 | 1 | 1 | 3 |
| hsa-mir-182-5p | LSM14A | 1 | 1 | 1 | 3 |
| hsa-mir-211-5p | HCAR2 | 1 | 1 | 1 | 3 |
| hsa-mir-192-5p | PRKD3 | 1 | 1 | 1 | 3 |
| hsa-mir-373-3p | PBK | 1 | 1 | 1 | 3 |
| hsa-mir-373-3p | MIXL1 | 1 | 1 | 1 | 3 |
| hsa-mir-223-3p | CYB5A | 1 | 1 | 1 | 3 |
| hsa-mir-373-3p | TNFAIP1 | 1 | 1 | 1 | 3 |
| hsa-mir-96-5p | EIF4EBP2 | 1 | 1 | 1 | 3 |
| hsa-mir-211-5p | M6PR | 1 | 1 | 1 | 3 |
| hsa-mir-223-3p | LMO2 | 1 | 1 | 1 | 3 |
| hsa-mir-211-5p | PHF13 | 1 | 1 | 1 | 3 |
| hsa-mir-150-5p | ZNF347 | 1 | 1 | 1 | 3 |
| hsa-mir-182-5p | TCEAL7 | 1 | 1 | 1 | 3 |
| hsa-mir-96-5p | MAP3K3 | 1 | 1 | 1 | 3 |
| hsa-mir-210-3p | FGFRL1 | 1 | 1 | 1 | 3 |
| hsa-mir-373-3p | PSD3 | 1 | 1 | 1 | 3 |
| hsa-mir-217 | EZH2 | 1 | 1 | 1 | 3 |
| hsa-mir-383-5p | SRSF2 | 1 | 1 | 1 | 3 |
| hsa-mir-96-5p | DDAH1 | 1 | 1 | 1 | 3 |
| hsa-mir-506-3p | PARP16 | 1 | 1 | 1 | 3 |
| hsa-mir-373-3p | SLC7A11 | 1 | 1 | 1 | 3 |
| hsa-mir-217 | KRAS | 1 | 1 | 1 | 3 |
| hsa-mir-223-3p | SMARCD1 | 1 | 1 | 1 | 3 |
| hsa-mir-31-5p | TBXA2R | 1 | 1 | 1 | 3 |
| hsa-mir-183-5p | DAP | 1 | 1 | 1 | 3 |
| hsa-mir-96-5p | APPL1 | 1 | 1 | 1 | 3 |
| hsa-mir-223-3p | TOX | 1 | 1 | 1 | 3 |
| hsa-mir-150-5p | PTGIS | 1 | 1 | 1 | 3 |
| hsa-mir-506-3p | SNX18 | 1 | 1 | 1 | 3 |
| hsa-mir-183-5p | PRRC1 | 1 | 1 | 1 | 3 |
| hsa-mir-192-5p | TMTC3 | 1 | 1 | 1 | 3 |
| hsa-mir-217 | DACH1 | 1 | 1 | 1 | 3 |
| hsa-mir-211-5p | ANKFY1 | 1 | 1 | 1 | 3 |
| hsa-mir-223-3p | NFIA | 1 | 1 | 1 | 3 |
| hsa-mir-211-5p | SLC39A9 | 1 | 1 | 1 | 3 |
| hsa-mir-192-5p | ZBTB34 | 1 | 1 | 1 | 3 |
| hsa-mir-373-3p | PLA2G12A | 1 | 1 | 1 | 3 |
| hsa-mir-31-5p | MZT1 | 1 | 1 | 1 | 3 |
| hsa-mir-182-5p | FGF9 | 1 | 1 | 1 | 3 |
| hsa-mir-211-5p | RAB22A | 1 | 1 | 1 | 3 |
| hsa-mir-223-3p | SP3 | 1 | 1 | 1 | 3 |
| hsa-mir-373-3p | CPT1A | 1 | 1 | 1 | 3 |
| hsa-mir-373-3p | ELK4 | 1 | 1 | 1 | 3 |
| hsa-mir-214-3p | AMER1 | 1 | 1 | 1 | 3 |
| hsa-mir-373-3p | MED17 | 1 | 1 | 1 | 3 |
| hsa-mir-211-5p | BCL2 | 1 | 1 | 1 | 3 |
| hsa-mir-211-5p | TPPP | 1 | 1 | 1 | 3 |
| hsa-mir-192-5p | DDX50 | 1 | 1 | 1 | 3 |
| hsa-mir-506-3p | SFT2D3 | 1 | 1 | 1 | 3 |
| hsa-mir-217 | LMLN | 1 | 1 | 1 | 3 |
| hsa-mir-31-5p | PRKCE | 1 | 1 | 1 | 3 |
| hsa-mir-214-3p | FLOT2 | 1 | 1 | 1 | 3 |
| hsa-mir-214-3p | FGFR1 | 1 | 1 | 1 | 3 |
| hsa-mir-373-3p | KPNA2 | 1 | 1 | 1 | 3 |
| hsa-mir-223-3p | POLR3G | 1 | 1 | 1 | 3 |
| hsa-mir-373-3p | CCSAP | 1 | 1 | 1 | 3 |
| hsa-mir-373-3p | IRAK4 | 1 | 1 | 1 | 3 |
| hsa-mir-383-5p | ADSS | 1 | 1 | 1 | 3 |
| hsa-mir-214-3p | CDC42SE1 | 1 | 1 | 1 | 3 |
| hsa-mir-211-5p | SERINC3 | 1 | 1 | 1 | 3 |
| hsa-mir-96-5p | NHLRC3 | 1 | 1 | 1 | 3 |
| hsa-mir-373-3p | MICA | 1 | 1 | 1 | 3 |
| hsa-mir-373-3p | RAD23B | 1 | 1 | 1 | 3 |
| hsa-mir-31-5p | ARF1 | 1 | 1 | 1 | 3 |
| hsa-mir-373-3p | TIMM17A | 1 | 1 | 1 | 3 |
| hsa-mir-183-5p | ZEB1 | 1 | 1 | 1 | 3 |
| hsa-mir-373-3p | RAB22A | 1 | 1 | 1 | 3 |
| hsa-mir-183-5p | ARFGAP2 | 1 | 1 | 1 | 3 |
| hsa-mir-192-5p | WDR44 | 1 | 1 | 1 | 3 |
| hsa-mir-96-5p | SCARB1 | 1 | 1 | 1 | 3 |
| hsa-mir-183-5p | SH3D19 | 1 | 1 | 1 | 3 |
| hsa-mir-150-5p | TLDC1 | 1 | 1 | 1 | 3 |
| hsa-mir-373-3p | LIMA1 | 1 | 1 | 1 | 3 |
| hsa-mir-214-3p | PIM1 | 1 | 1 | 1 | 3 |
| hsa-mir-150-5p | ZNF460 | 1 | 1 | 1 | 3 |
| hsa-mir-373-3p | KLF3 | 1 | 1 | 1 | 3 |
| hsa-mir-373-3p | TAOK1 | 1 | 1 | 1 | 3 |
| hsa-mir-217 | NR4A2 | 1 | 1 | 1 | 3 |
| hsa-mir-96-5p | TRIB3 | 1 | 1 | 1 | 3 |
| hsa-mir-96-5p | PROK2 | 1 | 1 | 1 | 3 |
| hsa-mir-192-5p | MSN | 1 | 1 | 1 | 3 |
| hsa-mir-192-5p | L2HGDH | 1 | 1 | 1 | 3 |
| hsa-mir-214-3p | GABARAP | 1 | 1 | 1 | 3 |
| hsa-mir-192-5p | TCF7 | 1 | 1 | 1 | 3 |
| hsa-mir-506-3p | CD151 | 1 | 1 | 1 | 3 |
| hsa-mir-217 | GPC5 | 1 | 1 | 1 | 3 |
| hsa-mir-373-3p | FAM102B | 1 | 1 | 1 | 3 |
| hsa-mir-182-5p | EVI5 | 1 | 1 | 1 | 3 |
| hsa-mir-192-5p | DYRK1A | 1 | 1 | 1 | 3 |
| hsa-mir-373-3p | YOD1 | 1 | 1 | 1 | 3 |
| hsa-mir-96-5p | STK17B | 1 | 1 | 1 | 3 |
| hsa-mir-214-3p | ZNRF1 | 1 | 1 | 1 | 3 |
| hsa-mir-373-3p | CLIP4 | 1 | 1 | 1 | 3 |
| hsa-mir-373-3p | MKNK2 | 1 | 1 | 1 | 3 |
| hsa-mir-210-3p | MDGA1 | 1 | 1 | 1 | 3 |
| hsa-mir-150-5p | AHI1 | 1 | 1 | 1 | 3 |
| hsa-mir-183-5p | IDH2 | 1 | 1 | 1 | 3 |
| hsa-mir-192-5p | TMPO | 1 | 1 | 1 | 3 |
| hsa-mir-383-5p | VEGFA | 1 | 1 | 1 | 3 |
| hsa-mir-211-5p | KLHL40 | 1 | 1 | 1 | 3 |
| hsa-mir-373-3p | ATAD2 | 1 | 1 | 1 | 3 |
| hsa-mir-373-3p | CFL2 | 1 | 1 | 1 | 3 |
| hsa-mir-373-3p | TMEM19 | 1 | 1 | 1 | 3 |
| hsa-mir-214-3p | TFAP2C | 1 | 1 | 1 | 3 |
| hsa-mir-182-5p | PLEKHA8 | 1 | 1 | 1 | 3 |
| hsa-mir-223-3p | F3 | 1 | 1 | 1 | 3 |
| hsa-mir-373-3p | CREBRF | 1 | 1 | 1 | 3 |
| hsa-mir-31-5p | FOXD4L1 | 1 | 1 | 1 | 3 |
| hsa-mir-373-3p | LEFTY2 | 1 | 1 | 1 | 3 |
| hsa-mir-373-3p | PTPDC1 | 1 | 1 | 1 | 3 |
| hsa-mir-31-5p | GTF2E1 | 1 | 1 | 1 | 3 |
| hsa-mir-150-5p | AIFM2 | 1 | 1 | 1 | 3 |
| hsa-mir-96-5p | SLC1A1 | 1 | 1 | 1 | 3 |
| hsa-mir-373-3p | CUL3 | 1 | 1 | 1 | 3 |
| hsa-mir-214-3p | NUFIP2 | 1 | 1 | 1 | 3 |
| hsa-mir-211-5p | NUAK1 | 1 | 1 | 1 | 3 |
| hsa-mir-210-3p | SERTM1 | 1 | 1 | 1 | 3 |
| hsa-mir-96-5p | PRKCE | 1 | 1 | 1 | 3 |
| hsa-mir-31-5p | HIF1AN | 1 | 1 | 1 | 3 |
| hsa-mir-211-5p | FOXC1 | 1 | 1 | 1 | 3 |
| hsa-mir-192-5p | STX7 | 1 | 1 | 1 | 3 |
| hsa-mir-506-3p | PTBP1 | 1 | 1 | 1 | 3 |
| hsa-mir-192-5p | CUL3 | 1 | 1 | 1 | 3 |
| hsa-mir-214-3p | CS | 1 | 1 | 1 | 3 |
| hsa-mir-373-3p | HABP4 | 1 | 1 | 1 | 3 |
| hsa-mir-373-3p | ZNF532 | 1 | 1 | 1 | 3 |
| hsa-mir-183-5p | KIF2A | 1 | 1 | 1 | 3 |
| hsa-mir-373-3p | KREMEN1 | 1 | 1 | 1 | 3 |
| hsa-mir-182-5p | TP53INP1 | 1 | 1 | 1 | 3 |
| hsa-mir-96-5p | PRDM16 | 1 | 1 | 1 | 3 |
| hsa-mir-214-3p | CTNNB1 | 1 | 1 | 1 | 3 |
| hsa-mir-373-3p | TFAP4 | 1 | 1 | 1 | 3 |
| hsa-mir-183-5p | ARHGAP21 | 1 | 1 | 1 | 3 |
| hsa-mir-214-3p | SCAMP4 | 1 | 1 | 1 | 3 |
| hsa-mir-214-3p | SOCS5 | 1 | 1 | 1 | 3 |
| hsa-mir-183-5p | KLHL23 | 1 | 1 | 1 | 3 |
| hsa-mir-223-3p | MEF2C | 1 | 1 | 1 | 3 |
| hsa-mir-182-5p | THBS1 | 1 | 1 | 1 | 3 |
| hsa-mir-182-5p | ULBP2 | 1 | 1 | 1 | 3 |
| hsa-mir-506-3p | PTBP3 | 1 | 1 | 1 | 3 |
| hsa-mir-183-5p | SRSF2 | 1 | 1 | 1 | 3 |
| hsa-mir-96-5p | PPP1R9B | 1 | 1 | 1 | 3 |
| hsa-mir-211-5p | SLC43A1 | 1 | 1 | 1 | 3 |
| hsa-mir-214-3p | TBPL1 | 1 | 1 | 1 | 3 |
| hsa-mir-182-5p | NUFIP2 | 1 | 1 | 1 | 3 |
| hsa-mir-150-5p | PDCD4 | 1 | 1 | 1 | 3 |
| hsa-mir-183-5p | FOXN2 | 1 | 1 | 1 | 3 |
| hsa-mir-506-3p | TMEM41A | 1 | 1 | 1 | 3 |
| hsa-mir-373-3p | ULK1 | 1 | 1 | 1 | 3 |
| hsa-mir-373-3p | ARID4B | 1 | 1 | 1 | 3 |
| hsa-mir-192-5p | RIC8B | 1 | 1 | 1 | 3 |
| hsa-mir-183-5p | NUFIP2 | 1 | 1 | 1 | 3 |
| hsa-mir-214-3p | NFIC | 1 | 1 | 1 | 3 |
| hsa-mir-211-5p | RAB10 | 1 | 1 | 1 | 3 |
| hsa-mir-192-5p | FAM199X | 1 | 1 | 1 | 3 |
| hsa-mir-183-5p | CELF1 | 1 | 1 | 1 | 3 |
| hsa-mir-96-5p | ADCY6 | 1 | 1 | 1 | 3 |
| hsa-mir-373-3p | LUC7L2 | 1 | 1 | 1 | 3 |
| hsa-mir-182-5p | FOXF2 | 1 | 1 | 1 | 3 |
| hsa-mir-96-5p | SLC25A25 | 1 | 1 | 1 | 3 |
| hsa-mir-211-5p | HOXC8 | 1 | 1 | 1 | 3 |
| hsa-mir-31-5p | ABCB9 | 1 | 1 | 1 | 3 |
| hsa-mir-373-3p | DUSP2 | 1 | 1 | 1 | 3 |
| hsa-mir-182-5p | NR3C1 | 1 | 1 | 1 | 3 |
| hsa-mir-96-5p | SIN3B | 1 | 1 | 1 | 3 |
| hsa-mir-150-5p | MBD6 | 1 | 1 | 1 | 3 |
| hsa-mir-182-5p | RECK | 1 | 1 | 1 | 3 |
| hsa-mir-373-3p | CMTM4 | 1 | 1 | 1 | 3 |
| hsa-mir-183-5p | NR3C1 | 1 | 1 | 1 | 3 |
| hsa-mir-31-5p | ZNF805 | 1 | 1 | 1 | 3 |
